# Dichotomous Dynamics in E-I Networks with Strongly and Weakly Intra-connected Inhibitory Neurons

**DOI:** 10.3389/fncir.2017.00104

**Published:** 2017-12-13

**Authors:** Scott Rich, Michal Zochowski, Victoria Booth

**Affiliations:** ^1^Applied and Interdisciplinary Mathematics, University of Michigan, Ann Arbor, MI, United States; ^2^Department of Physics and Biophysics, University of Michigan, Ann Arbor, MI, United States; ^3^Department of Mathematics and Anesthesiology, University of Michigan, Ann Arbor, MI, United States

**Keywords:** PING, E-I network, interneurons, heterogeneity, oscillations, rhythms, computational model

## Abstract

The interconnectivity between excitatory and inhibitory neural networks informs mechanisms by which rhythmic bursts of excitatory activity can be produced in the brain. One such mechanism, Pyramidal Interneuron Network Gamma (PING), relies primarily upon reciprocal connectivity between the excitatory and inhibitory networks, while also including intra-connectivity of inhibitory cells. The causal relationship between excitatory activity and the subsequent burst of inhibitory activity is of paramount importance to the mechanism and has been well studied. However, the role of the intra-connectivity of the inhibitory network, while important for PING, has not been studied in detail, as most analyses of PING simply assume that inhibitory intra-connectivity is strong enough to suppress subsequent firing following the initial inhibitory burst. In this paper we investigate the role that the strength of inhibitory intra-connectivity plays in determining the dynamics of PING-style networks. We show that networks with weak inhibitory intra-connectivity exhibit variations in burst dynamics of both the excitatory and inhibitory cells that are not obtained with strong inhibitory intra-connectivity. Networks with weak inhibitory intra-connectivity exhibit excitatory rhythmic bursts with weak excitatory-to-inhibitory synapses for which classical PING networks would show no rhythmic activity. Additionally, variations in dynamics of these networks as the excitatory-to-inhibitory synaptic weight increases illustrates the important role that consistent pattern formation in the inhibitory cells serves in maintaining organized and periodic excitatory bursts. Finally, motivated by these results and the known diversity of interneurons, we show that a PING-style network with two inhibitory subnetworks, one strongly intra-connected and one weakly intra-connected, exhibits organized and periodic excitatory activity over a larger parameter regime than networks with a homogeneous inhibitory population. Taken together, these results serve to better articulate the role of inhibitory intra-connectivity in generating PING-like rhythms, while also revealing how heterogeneity amongst inhibitory synapses might make such rhythms more robust to a variety of network parameters.

## Introduction

The importance of inhibitory interneurons in driving and modulating rhythmic electrical activity is well established in a variety of brain regions, including the hippocampus (Traub et al., 1998; Kopell et al., 2000; Bartos et al., 2007; Aton et al., 2013) and the cortex (Desimone and Duncan, 1995; Luck et al., 1997; Reynolds et al., 1999; Fries, 2005; Bosman et al., 2012). Computational studies have identified mechanisms by which inhibition is the impetus behind rhythmic and synchronous dynamics for strictly inhibitory neural networks and for networks with both excitatory and inhibitory neurons (hereafter referred to as E-I networks). For strictly inhibitory neural networks, Interneuron Network Gamma (ING) is perhaps the most well-known mechanism underlying inhibitory synchrony (White et al., 1998; Whittington et al., 2000; Kopell et al., 2010; Wang, 2010), although additional studies have shown that other mechanisms and forms of rhythmic dynamics may arise due to changing the intrinsic cellular properties or connectivity density in these networks (Vreeswijk et al., 1994; Hansel et al., 1995; Achuthan and Canavier, 2009; Ladenbauer et al., 2012; Rich et al., 2016; Viriyopase et al., 2016).

In E-I networks, the Pyramidal Interneuron Network Gamma (PING) mechanism can generate rhythmic dynamics. Experimental results have implicated interactions between excitatory and inhibitory neurons in this rhythm generation (Whittington et al., 1995), leading to computational studies probing the required properties of these interactions for PING rhythms (Traub et al., 1997; Ermentrout and Kopell, 1998; Whittington et al., 2000; Kopell et al., 2010). Summarized, the PING mechanism states that synchronous, rhythmic dynamics of both the excitatory and inhibitory cell populations can be generated if the inhibitory cells spike only in response to excitatory cell activity, if excitatory cell activity quickly induces a synchronous inhibitory population burst, and if the inhibitory burst suppresses all excitatory cells. These requirements ensure that bursts of inhibitory activity directly follow excitatory cell activity, and this inhibition then suppresses the excitatory cells for a sufficient duration so that all excitatory cells are set to the same point in their firing cycle, so that upon the release of inhibition their next action potentials occur in close temporal proximity, resulting in synchronous firing.

This conceptual PING mechanism has led to research probing the robustness of the mechanism to various forms of randomness and heterogeneity that are likely to occur in the brain. Such studies have investigated the role of sparse and heterogeneous connectivity in PING rhythm formation (Börgers and Kopell, 2003), the role of the strength of interconnectivity between the excitatory and inhibitory neurons in eliciting PING rhythms (Börgers et al., 2012), the effects of noise on these rhythms (Börgers and Kopell, 2005), changes caused by changing the properties of the inhibitory neurons from Type I to Type II (these classifications are defined below) (Borgers and Walker, 2013), and various effects of adaptation currents in the cell models (Olufsen et al., 2003; Krupa et al., 2014). These studies focus primarily on the interconnectivity between the excitatory and inhibitory cell populations, which according to the PING mechanism is the paramount impetus behind rhythmic activity.

Little work has been done, however, to analyze the role of the intra-connectivity of the inhibitory cell population in affecting the dynamics of E-I networks. Indeed, as classically articulated this inhibitory intra-connectivity is not necessary for PING rhythm generation, but is typically included in the network structure as motivated by numerous experimental studies showing that interneurons tend to be highly connected (Markram et al., 2004; Mody and Pearce, 2004; Tateno and Robinson, 2007; Karson et al., 2009; Ferguson et al., 2013; Perrenoud et al., 2013). Thus, most of the computational studies above assume some level of strong synaptic coupling amongst the interneurons, which serves to “slow down” and help prevent disorganized firing of the inhibitory cells that can disrupt synchronous firing of the excitatory cells (Börgers and Kopell, 2005).

Given the multitude of types of interneurons identified in brain regions where PING rhythms are thought to occur, such as the hippocampus (Buhl et al., 1994; Klausberger et al., 2003) and cortex (Gonchar and Burkhalter, 1997; Gibson et al., 1999; Beierlein et al., 2000, 2003; Barthó et al., 2004; Somogyi and Klausberger, 2005; Klausberger and Somogyi, 2008), along with the known connectivity of many of these interneurons with excitatory pyramidal cells as modeled in an E-I network (Whittington and Traub, 2003), a closer analysis of the role of inhibitory intra-connectivity in PING-driven dynamics will paint a more complete picture of how such rhythmic activity might arise in the brain. In this paper, we investigate in detail the role that the strength of inhibitory intra-connectivity (I-I connectivity) plays in dictating the burst dynamics of excitatory cells in E-I networks. While some differences in bursting dynamics that arise from weakening the inhibitory intra-connectivity to values well below that typically studied in the PING literature, such as inhibitory spike doublets, have been identified previously (Börgers and Kopell, 2005), we find that these differences can have important effects on the spiking properties of the excitatory network.

Our results show that, when interneurons have Type I firing properties (similar to those often exhibited by the ubiquitous fast-spiking PV+ interneuron, Ferguson et al., 2013), there is a distinct difference in rhythmic synchronous dynamics when the strength of I-I connectivity varies from weak to strong. Here, we define Type I neurons as those that exhibit a steep current-frequency (IF) curve with an arbitrarily low firing frequency and a Phase Response Curve (PRC) exhibiting only phase advances in response to a brief, excitatory current pulses (Brown et al., 2004; Tateno et al., 2004; Wang, 2010). We focus on networks with Type I interneurons given the preponderance of PV+ interneurons in the hippocampus and cortex, and also because a majority of PING literature uses inhibitory neuron models with these properties.

When the I-I connectivity is strong, our results closely mirror those of most analyses of PING rhythms. The dynamics of these networks not only include synchrony amongst the excitatory cells, but also lead to organized spike timing within the excitatory bursts, consistent cell participation in each burst, and consistent periodicity of the bursts. We classify these dynamics as having “low variability” to differentiate them from networks that exhibit synchronous bursting but without this additional spike organization. When the excitatory to inhibitory connectivity (E-I connectivity) is very weak, however, networks with this strong intra-connectivity amongst the inhibitory cells exhibit asynchonous excitatory cell activity, as predicted by the PING mechanism.

On the other hand, when I-I connectivity is weak, E-I networks exhibit alternate dynamics, highlighting the importance of inhibitory cell patterning in dictating excitatory cell dynamics. In this case, synchronous excitatory activity is exhibited at very low values of the E-I connectivity strength, seemingly in opposition to one of the key requirements of PING theory. As E-I connectivity strength increases, these networks tend to exhibit disorganized inhibitory cell firing that follows inhibitory bursts, which in turn leads to degradation of excitatory cell synchrony. Namely, excitatory bursts tend to be disorganized, exhibit variability in the number of cells participating in each burst, and do not exhibit a consistent inter-burst interval.

To determine dependence on inhibitory cellular properties, we additionally examine how changing I-I connectivity affects networks where the interneurons have Type II firing properties. Here we define Type II neurons as those that exhibit a more shallow IF curve with a minimum non-zero firing frequency and a PRC exhibiting regions of phase delay and advance in response to brief, excitatory current pulses (Brown et al., 2004; Wang, 2010). We find that changes to the I-I connectivity in E-I networks do not significantly alter dynamics of the overall network when the interneurons are modeled as Type II. This corresponds with our previous work which shows that, unlike strictly inhibitory networks with Type I neurons, such networks containing Type II neurons with and without an M-type slow potassium current do not show significant changes in the propensity for synchrony as the connectivity density, which roughly corresponds with the overall strength of inhibitory signaling in the network, changes (Rich et al., 2016).

Our analysis indicates that synchronous, rhythmic, PING activity in E-I networks consisting of Type I interneurons is sensitive to I-I connectivity strength. Specifically, weak inhibitory intra-connectivity allows well-ordered synchronous excitatory activity primarily for low values of the E-I synaptic weight, while networks with strong inhibitory intra-connectivity exhibit such activity for high values of the E-I synaptic weight. This dichotomy motivates the investigation of a network architecture that preserves the advantages of both types of networks, effectively expanding the parameter regime at which PING-like rhythms can be achieved. An E-I network with two inhibitory subnetworks, one weakly intra-connected and one strongly intra-connected, achieves this goal, providing a potential mechanism by which such rhythms can be generated in the brain in a more robust fashion. Numerous studies provide support for the existence of this type of network topology in the brain, where multiple populations of interneurons synapse onto the same excitatory pyramidal cells while connectivity between inhibitory interneurons consists almost exclusively of synapses between similar interneurons (Gibson et al., 1999; Beierlein et al., 2003; Klausberger et al., 2003; Somogyi and Klausberger, 2005; Wang et al., 2011).

Taken together, these results serve to expand upon our understanding of PING-like rhythms in E-I networks by revealing the important, but often overlooked, role that inhibitory intra-connectivity and inhibitory cell dynamics play in governing the overall network dynamics.

## Methods

### Neuron models

We constructed E-I networks consisting of excitatory neurons with Type I membrane properties and inhibitory neurons with Type I or Type II membrane properties. The Type I neuron model, in the Hodgkin-Huxley formalism, mirrors the fast-spiking properties of PV+ interneurons as well as properties of Type I cortical pyramidal neurons (Stiefel et al., 2008; Fink et al., 2011). The equations governing this model are:

(1) $$\begin{array}{l} \frac{dV}{dt}=-g_{Na}m_{\infty }^{3}h\left(V-E_{Na}\right)-g_{K_{d}}n^{4}\left(V-E_{K}\right)\\ \;-g_{K_{s}}z\left(V-E_{K}\right)-g_{L}\left(V-E_{L}\right)+I_{app}-I_{syn} \end{array}$$

(2) $$\begin{array}{l} \frac{dX}{dt}=\frac{X_{\infty }\left(V\right)-X}{\tau_{X}\left(V\right)}\text{for}X=h,n,z \end{array}$$

(3) $$\begin{array}{l} m_{\infty }\left(V\right)=\frac{1}{1+e^{\left(-V-30/9.5\right)}} \end{array}$$

(4) $$\begin{array}{l} h_{\infty }\left(V\right)=\frac{1}{1+e^{\left(V+53/7.0\right)}} \end{array}$$

(5) $$\begin{array}{l} n_{\infty }\left(V\right)=\frac{1}{1+e^{\left(-V-30/10\right)}} \end{array}$$

(6) $$\begin{array}{l} z_{\infty }\left(V\right)=\frac{1}{1+e^{\left(-V-39/5\right)}} \end{array}$$

(7) $$\begin{array}{l} \tau_{h}\left(V\right)=0.37+\frac{2.78}{1+e^{\left(V+40.5\right)/6}} \end{array}$$

(8) $$\begin{array}{l} \tau_{n}\left(V\right)=0.37+\frac{1.85}{1+e^{\left(V+27\right)/15}} \end{array}$$

(9) $$\begin{array}{l} \tau_{z}\left(V\right)=75 \end{array}$$

*V* represents the membrane voltage in [mV], while *m, n, h* and *z* represent the unitless gating variables of the ionic current conductances. *I*_*app*_ signifies the external applied current to the neuron (described below), in [μA/cm^2^], while *I*_*syn*_ describes the synaptic current input to the cell from the network (described below), also with units of [μA/cm^2^]. *E*_*Na*_, *E*_*K*_*s*__, *E*_*K*_*d*__ and *E*_*L*_ are the reversal potentials and *g*_*Na*_, *g*_*K*_*s*__, *g*_*K*_*d*__ and *g*_*L*_ are the maximum conductances, with *Na* symbolizing sodium, *K* symbolizing potassium, and *L* symbolizing the leak current. *K*_*d*_ refers to the delayed rectifier potassium current, while *K*_*s*_ refers to the slow M-type potassium current (which is inactive when this model simulates the Type I neuron used here). In this model the reversal potentials are *E*_*Na*_ = 55 mV, *E*_*K*_ = −90 mV, *E*_*L*_ = −60 mV, while the maximum conductances are *g*_*Na*_ = 24 mS/cm^2^, *g*_*K*_*d*__ = 3 mS/cm^2^, *g*_*K*_*s*__ = 0 mS/cm^2^ and *g*_*L*_=0.02 mS/cm^2^.

While the equations for the Type I neuron were initially developed to model a cortical pyramidal neuron modulated by acetylcholine, the properties of this neuron when *g*_*K*_*s*__ = 0 closely mirror those of fast-spiking Type I interneurons (for instance, the PV interneurons modeled by Ferguson et. al.).

Networks in which the interneurons were replaced with a Type II neuron with adaptation used the same model equations as the Type I case, but with the value of *g*_*K*_*s*__ changed to 1.5. This activates the slow M-type potassium current, which in turn changes the neuron properties to Type II and imbues the neurons with properties similar to interneurons like the OLM and SOM cells (Saraga et al., 2003; Markram et al., 2004; Lawrence et al., 2006; Cutsuridis et al., 2010; Cutsuridis and Hasselmo, 2012; Perrenoud et al., 2013).

For comparison purposes, we also study networks with interneurons that are Type II without the presence of an adaptation current. These neurons were modeled with the classic Hodgkin-Huxley equations (Hodgkin and Huxley, 1952; Ermentrout and Terman, 2010):

(10) $$\begin{array}{l} \frac{dV}{dt}=-g_{Na}m^{3}h\left(V-E_{Na}\right)-g_{K}n^{4}\left(V-E_{K}\right)-g_{L}\left(V-E_{L}\right)\\ \;+I_{app}-I_{syn} \end{array}$$

(11) $$\begin{array}{l} \frac{dX}{dt}=\alpha_{X}\left(V\right)\left(1-X\right)-\beta_{X}\left(V\right)X,\text{for}X=m,h,n \end{array}$$

(12) $$\begin{array}{l} \alpha_{m}\left(V\right)=-0.1\left(\frac{V+40}{e^{-\left(V+40\right)/10}-1}\right) \end{array}$$

(13) $$\begin{array}{l} \beta_{m}\left(V\right)=4e^{-\left(V+65\right)/18} \end{array}$$

(14) $$\begin{array}{l} \alpha_{h}\left(V\right)=0.07e^{-\left(V+65\right)/20} \end{array}$$

(15) $$\begin{array}{l} \beta_{h}\left(V\right)=\frac{1.0}{e^{-\left(V+35\right)/10}+1} \end{array}$$

(16) $$\begin{array}{l} \alpha_{n}\left(V\right)=-0.01\left(\frac{V+55}{e^{-\left(V+55\right)/10}-1}\right) \end{array}$$

(17) $$\begin{array}{l} \beta_{n}\left(V\right)=1.25e^{-\left(V+65\right)/80} \end{array}$$

The variables here signify the same quantities as in the above equations for Type I neurons. In this model these constants are set at *E*_*Na*_ = 50 mV, *E*_*K*_ = −77 mV, *E*_*L*_ = −54.4 mV, *g*_*Na*_ = 120 mS/cm^2^, *g*_*K*_ = 36 mS/cm^2^ and *g*_*L*_ = 0.3 mS/cm^2^.

### Network structure

**Figure 1:**
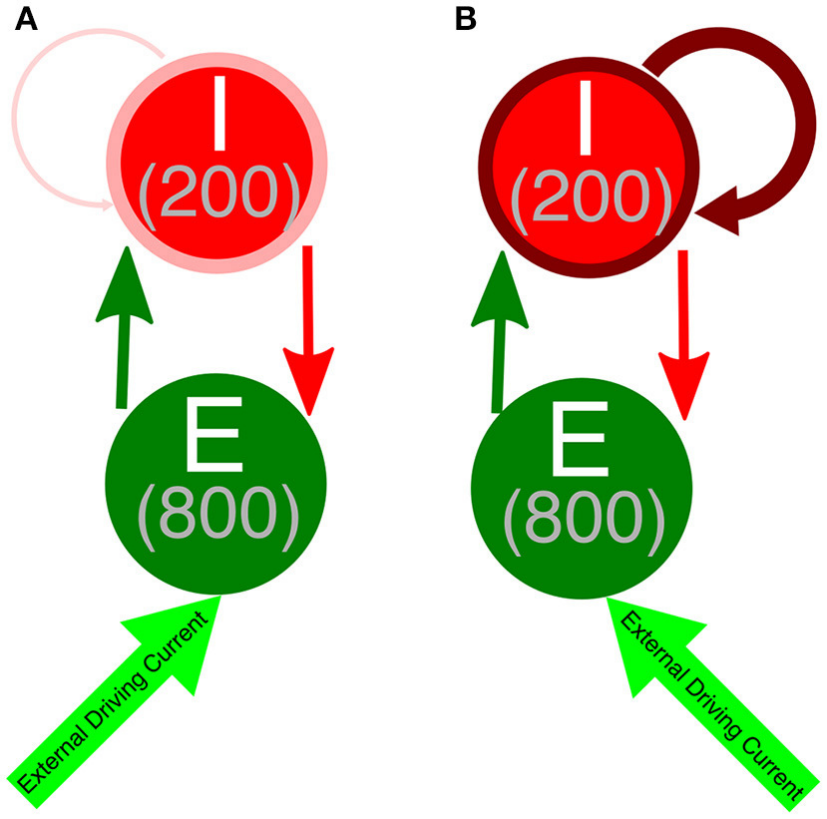
Network diagram of E-I networks. **(A)** Connectivity in an E-I network with a weakly connected inhibitory subnetwork. Thin, light red arrow symbolizes the weak intraconnectivity between inhibitory interneurons. **(B)** Connectivity in an E-I network with a strongly connected inhibitory subnetwork. Thick, dark red arrow symbolizes the strong intraconnectivity between inhibitory interneurons. In both diagrams, the dark green arrow symbolizes E-I synapses, while the red arrow indicates I-E synapses.

We performed simulations of E-I networks consisting of 1,000 neurons, 800 of which are excitatory and 200 of which are inhibitory. Excitatory neurons receive an external driving current (described below) and also receive inhibition from the inhibitory cells, where each inhibitory cell has a 50% chance to synapse onto a given excitatory cell. Inhibitory neurons receive an external current (described below) depending upon their cell type in order to ensure they do not fire in the absence of input from the excitatory cells and are near their firing threshold. Inhibitory neurons are driven by the excitatory cell population, as each excitatory cell has a 50% chance to synapse onto a given inhibitory cell. Additionally, inhibitory neurons receive inhibition from within the inhibitory network, as each inhibitory neuron has a 30% chance to synapse onto a given, different inhibitory cell. The choice of this connectivity density is motivated by evidence for this level of intraconnectivity amongst interneurons in the hippocampus (Ascoli and Atkeson, 2005; Viriyopase et al., 2016). Diagramatic representations of these networks with strong and weak inhibitory intra-connectivity are shown in Figure 1.

Cell heterogeneity was implemented by varying the external input current, *I*_*app*_, to each excitatory neuron. The input currents were selected from a uniform distribution centered on the current (*I*_*A*_) that would impart an average intrinsic cell firing frequency to an isolated neuron. For excitatory cells, we chose the driving currents uniformly from the distribution [0.9*I*_*A*_, 1.1*I*_*A*_]. *I*_*A*_ was varied for the excitatory cells in order to study the effects of their intrinsic frequency.

Type I inhibitory cells were given a small external hyperpolarizing current to ensure that the neurons would not fire spontaneously, given that this neuron model exhibits slow, spontaneous firing with no external current. Heterogeneity was implemented in this hyperpolarizing (i.e., negative) current similar to the excitatory cells to impart some degree of heterogeneity to the inhibitory population: the external hyperpolarizing current for each interneuron was chosen uniformly from the distribution [1.05*I*_*A*_, 0.95*I*_*A*_]. Here *I*_*A*_ is chosen to be −0.2 μ/cm^2^ so that all interneurons will not fire action potentials without input from the excitatory cells. This external hyperpolarizing current was not needed when the inhibitory cells were modeled using either Type II formalism, as those model neurons will not fire spontaneously.

We modeled synapses using a double exponential profile of the form:

(18) $$\begin{array}{l} I_{syn}\left(t\right)=g_{syn}\left(V-E_{syn}\right)\left(\underset{\begin{array}{c} s_{i} \end{array}}{\sum }e^{-\left(t-s_{i}\right)/\tau_{d}}-e^{-\left(t-s_{i}\right)/\tau_{r}}\right) \end{array}$$

where *g*_*syn*_ is the maximum conductance, *V* is the membrane voltage of the post-synaptic neuron, *E*_*syn*_ is the reversal potential of the synaptic current, *s*_*i*_ are the times of all pre-synaptic spikes occurring before the current time *t* in ms, and τ_*d*_ and τ_*r*_ are the synaptic decay and the synaptic rise time constants, respectively (in ms). *E*_*syn*_ is set at −75 mV for inhibitory synapses and 0 mV for excitatory synapses. τ_*r*_ is set at 0.2 ms for all synapses, while τ_*d*_ is set at 3.0 ms for excitatory synapses and 5.5 ms for inhibitory synapses. For I-E synapses, *g*_*syn*_ = 0.003 mS/cm^2^, while the synaptic weight for I-I and E-I synapses is varied in the simulations.

Simulations of strictly inhibitory networks utilize the network connectivity described in our previous work (Rich et al., 2016), where 1,000 inhibitory neurons are randomly connected with a 30% connectivity density. Cell heterogeneity is implemented in these simulations by varying the external driving current to the inhibitory neurons in a method analagous to that used for the excitatory neurons in the E-I networks described above. Synapses are also modeled identically to those described above.

### Noise

To investigate effects on our results of noisy perturbations to the network, we ran simulations where Poisson trains of brief excitatory stimuli were given to the excitatory neurons in addition to their tonic driving currents and synaptic currents. At each time step, there is a probability *p* = 10^−3^ that a given excitatory cell receives an excitatory “kick.” These kicks are modeled with a temporal profile similar to the excitatory synaptic currents in the network and are thus of the form:

(19) $$\begin{array}{l} I_{noise_{i}}\left(t\right)=g_{noise}\left(e^{-\left(t-s_{i}\right)/\tau_{d}}-e^{-\left(t-s_{i}\right)/\tau_{r}}\right) \end{array}$$

where *s*_*i*_ is the time of the *i*th kick to the cell, τ_*d*_ and τ_*r*_ are the same as for excitatory synaptic currents, and *g*_*noise*_ is the amplitude of each kick. The times of the 10 most recent kicks are stored and contribute to the drive to the cell, such that:

(20) $$\begin{array}{l} I_{noise_{total}}\left(t\right)=g_{noise}\left(\underset{\begin{array}{c} \left(k-9\right)\leq i\leq k \end{array}}{\sum }e^{-\left(t-s_{i}\right)/\tau_{d}}-e^{-\left(t-s_{i}\right)/\tau_{r}}\right) \end{array}$$

where *k* is the total number of kicks to the given cell at time *t*. This term is added to the overall current balance equation of the given cell, such that:

(21) $$\begin{array}{l} \frac{dV}{dt}=I_{ionic}+I_{app}-I_{syn}+I_{noise_{total}} \end{array}$$

where *I*_*ionic*_ denotes all terms besides *I*_*app*_ and *I*_*syn*_ found in Equations 1 and 10.

While the frequency of the noise was kept constant (i.e., *p* was the same for all simulations with noise), we varied *g*_*noise*_ in these simulations, as seen in **Figure 8**.

### Measures

We used several measures to quantify the dynamics of network activity. Foremost among them is the Synchrony Measure, an adaptation of a measure created by Golomb and Rinzel (1993, 1994) that quantifies the degree of spiking coincidence in the network. Briefly, the measure involves convolving a gaussian function with the time of each action potential for every cell to generate functions *V*_*i*_(*t*). The population averaged voltage *V*(*t*) is then defined as $V(t)=\frac{1}{N}\sum_{i=1}^{N}V_{i}\left(t\right)$, where *N* is the number of cells in the network. We further define the overall variance of the population averaged voltage σ and the variance of an individual neuron's voltage σ_*i*_ as:

(22) $$\begin{array}{l} \sigma =<V{\left(t\right)}^{2}>-<V\left(t\right){>}^{2} \end{array}$$

and

(23) $$\begin{array}{l} \sigma_{i}=<V_{i}{\left(t\right)}^{2}>-<V_{i}\left(t\right){>}^{2} \end{array}$$

where < · > indicates time averaging over the interval for which the measure is taken. The Synchrony Measure *S* is then defined as:

(24) $$\begin{array}{l} S=\frac{\sigma }{\frac{1}{N}\sum_{i=1}^{N}\sigma_{i}} \end{array}$$

The value *S* = 0 indicates completely asynchronous firing, while *S* = 1 corresponds to fully synchronous network activity.

The Synchrony Measure does not detect organization, or lack thereof, within each synchronous burst of network activity, or take into account the periodicity of the bursting dynamics. As such, we utilized three additional measures to quantify the organization and periodicity of excitatory bursting dynamics in our E-I networks.

Each of these three additional measures relied upon detecting instances of bursting activity within the excitatory network and identifying which neurons participated in the burst. To do this, the spike times of all the excitatory neurons in the network are convolved with a gaussian function to form a time trace of cumulative network activity. This trace is subsequently thresholded to determine the on and off times for every burst (*b*_*j*_ and *e*_*j*_, respectively). For each burst *j* we construct a binary vector that quantifies which neurons spiked during the burst, *v*_*j*_. If neuron *i* spiked during burst *j*, meaning it fires at a time *t*_*j*_ such that *b*_*j*_ ≤ *t*_*j*_ ≤ *e*_*j*_, we set *v*_*j*_(*i*) = 1, otherwise *v*_*j*_(*i*) = 0.

To analyze the organization of excitatory neurons within each network burst, we calculate the Variance of Neuron Order (*O*) in each simulation. For each burst of excitatory network activity in the last second of a given simulation (we term *k* the number of these bursts), the spike time of each firing neuron is detected and temporally ordered. We assign a value *O*_*i,j*_ for each neuron *i* in each burst *j* that conveys information on the ordering of the firing of neuron *i* within burst *j*. The firing order is normalized by the number of unique firing times in each excitatory burst and scaled between 1 and 100 such that the neurons that fire first have a value of 1 and the neurons that fire last (or not at all in a given burst) are given a value of 100. To calculate *O*, we take the standard deviation of the values *O*_*i,j*_ for 1 ≤ *j* ≤ *k* for each excitatory neuron *i*, and then average these 800 values to yield *O*. Low values of *O* indicate that neurons retain a predictable temporal ordering in each burst of activity; typically neurons with stronger external driving currents fire early and those with weaker driving currents fire later (where this variability in driving current is due to the implemented heterogeneity). High values of *O* indicate that the ordering of neuron firing within bursts is variable and thus the bursts do not retain significant ordering.

To analyze the consistency of cell participation in bursts, which we consider a measure of burst strength, we calculate the Variance of Active Cells (*A*) in each simulation. For each burst of excitatory network activity in the last second of a given simulation, the proportion of excitatory cells active in the burst is calculated, and *A* is defined as the standard deviation of these values. Low values of *A* indicate that each burst of excitatory network activity contains a similar proportion of the overall number of excitatory cells in the network, which in turn means that the strength of the excitatory signal sent to the inhibitory cell population is similar for each burst. High values of *A* indicate the number of active excitatory neurons varies from burst to burst; in turn, this causes significant variation in the strength of the excitatory signal sent to the inhibitory cell population.

Finally, to analyze the consistency of the periodic nature of excitatory network bursting activity, we calculate the Variance of Inter-burst Interval (*I*). The inter-burst interval between bursts of excitatory network activity is found for each burst occurring within the last second of a given simulation, and the coefficient of variation is calculated for these inter-burst intervals, giving the measure *I*. We note that we use the coefficient of variation in this measure because the standard deviation values vary with the average firing frequency of the bursts. Low values of *I* indicate that the network is periodic with very little variation in the timing between bursts of network activity. High values of *I* indicate that bursts of excitatory cell activity exhibit significant variability in their timing, which in turn means that the excitatory signal sent to the inhibitory cell population is not strictly periodic.

These three measures are combined together into one measure, dubbed the Variability Measure (*V*), to quantify the degree to which the excitatory network displays well-organized, periodic bursts typical of classic PING rhythmic activity. The measure is calculated thusly:

(25) $$\begin{array}{l} V=\sqrt{\left({Ō}^{2}+{Ā}^{2}+{Ī}^{2}\right)} \end{array}$$

where the bar indicates normalized values of each of the measures *O*, *A*, and *I* between 0 and 1.

Each measure is normalized by dividing by a maximal value that is slightly above the highest values of the measure typically achieved in networks exhibiting clear bursting activity amongst excitatory cells. When *S* < 0.2, which indicates that the network is asynchronous to the point that bursts of network activity do not occur, we artificially set the value of each normalized measure to 1. This ensures that the normalized values of each measure are 1 only in cases without clear bursting patterns. Via this algorithm, the normalized values of each measure are calculated as:

(26) $$\begin{array}{l} \text{for }X=O,A,I\text{:}\bar{X}=\left\{ \begin{array}{c} \frac{X}{X_{m}}\text{ if }S\geq 0.2\\ 1\text{ if }S<0.2\\ \; \end{array}\right. \end{array}$$

The Variability Measure thus takes the Euclidean Distance of these three measures when their values are scaled between 0 and 1, with 0 indicating minimal variability in the given metric and 1 indicating abnormally high variability or a lack of network bursts. The value *V* = 0 indicates a network in which there is no variability in the order of neurons within each burst, no variability of the number of neurons firing in each burst, and no variability in the inter-burst intervals. *V* will approach its maximum value of $\sqrt{3}$ in networks when high variability is detected by each of the three measures, and *V* will achieve its maximum value only if this variability is abnormally high in each measure or if no bursting activity is achieved by the network.

We also calculated the difference between the total excitatory and total inhibitory synaptic signaling (E-I Difference) in the inhibitory network. We calculate it as a mean difference between the total excitatory synaptic current and total inhibitory synaptic current received by the inhibitory cell population. As with our other measures, we analyze the final second of our simulations using the E-I Difference. We note that we utilized this measure instead of a ratio of excitatory and inhibitory synaptic current, as is common in E/I Balance measures, because such a ratio would tend toward infinity in networks with little or no inhibitory cell activity, which exist in our parameter space.

### Simulations

The code implementing these simulations was written in the C programming language and run on the University of Michigan's Flux cluster, a Linux-based high-performance computing cluster.

All simulations were run for 1,500 ms from random initial conditions for voltage and gating variables for each neuron. Possible initial conditions for *V* ranged between −62 and −22 mv, while the possible initial conditions for each gating variable ranged between 0.2 and 0.8.

Model equations were integrated using a fourth order Runge-Kutta technique. Spikes do not trigger synaptic current until 100 ms into the simulation to allow initial transients to decay.

Example raster plots shown throughout this paper are plotted such that the excitatory cells with the highest external driving current are given the lowest Neuron Index, and thus are plotted toward the bottom of the y-axis, while neurons with lowest external driving current are given the highest Neuron Index, and thus are plotted toward the top of the y-axis. This ordering of the excitatory cells was chosen to clearly illustrate the temporal organization of cells within a burst and does not reflect their location in the network.

All plots illustrating the various measures used to quantify network dynamics display the average of these scores over five independent simulations, where the measures are calculated over the last second of the simulation. The lone exception are the results shown in **Figure 10** for networks with Type II interneurons, for which only three repetitions were performed given the uniformity of the results.

## Results

**Figure 2:**
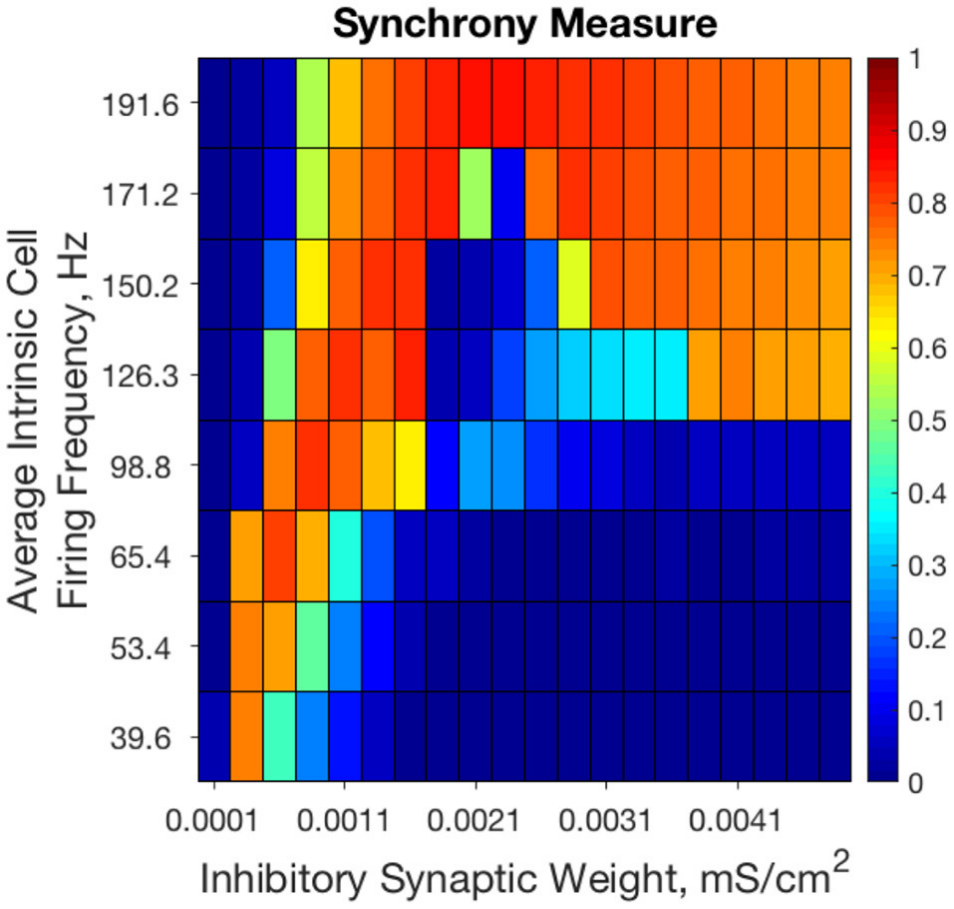
Randomly connected strictly inhibitory networks of Type I neurons with strong and weak inhibitory connectivity synchronize in divergent parameter regimes. Synchrony measure computed from dynamics of strictly inhibitory networks consisting of Type I neurons as synaptic weight (x-axis) and average intrinsic cell firing frequency (y-axis) are varied. Only networks with very weak inhibitory synaptic weight exhibit synchronous activity when average intrinsic cell firing frequency is low. Networks with stronger inhibitory synapses only synchronize when average intrinsic cell firing frequency is higher. Inhibitory synaptic weights stronger than those shown here simply continue the pattern of synchronous behavior shown for networks with an inhibitory synaptic weight above 0.0041 mS/cm^2^.

Previous results from our study of strictly inhibitory neural networks (Rich et al., 2016), combined with previous work in the field (White et al., 1998; Whittington et al., 2000; Kopell et al., 2010; Wang, 2010) show that synchronous bursting occurs in distinct parameter regimes for strongly connected and weakly connected networks of Type I neurons (see Figure 2). From random initial conditions, only networks with very weak inhibitory synaptic weight exhibit synchronous activity when average intrinsic cell firing frequency is low. As average intrinsic cell firing frequency increases, such networks exhibit asynchrony or weaker synchrony. In contrast, networks with stronger inhibitory synaptic weight synchronize only when average intrinsic cell firing frequency is high.

**Figure 3:**
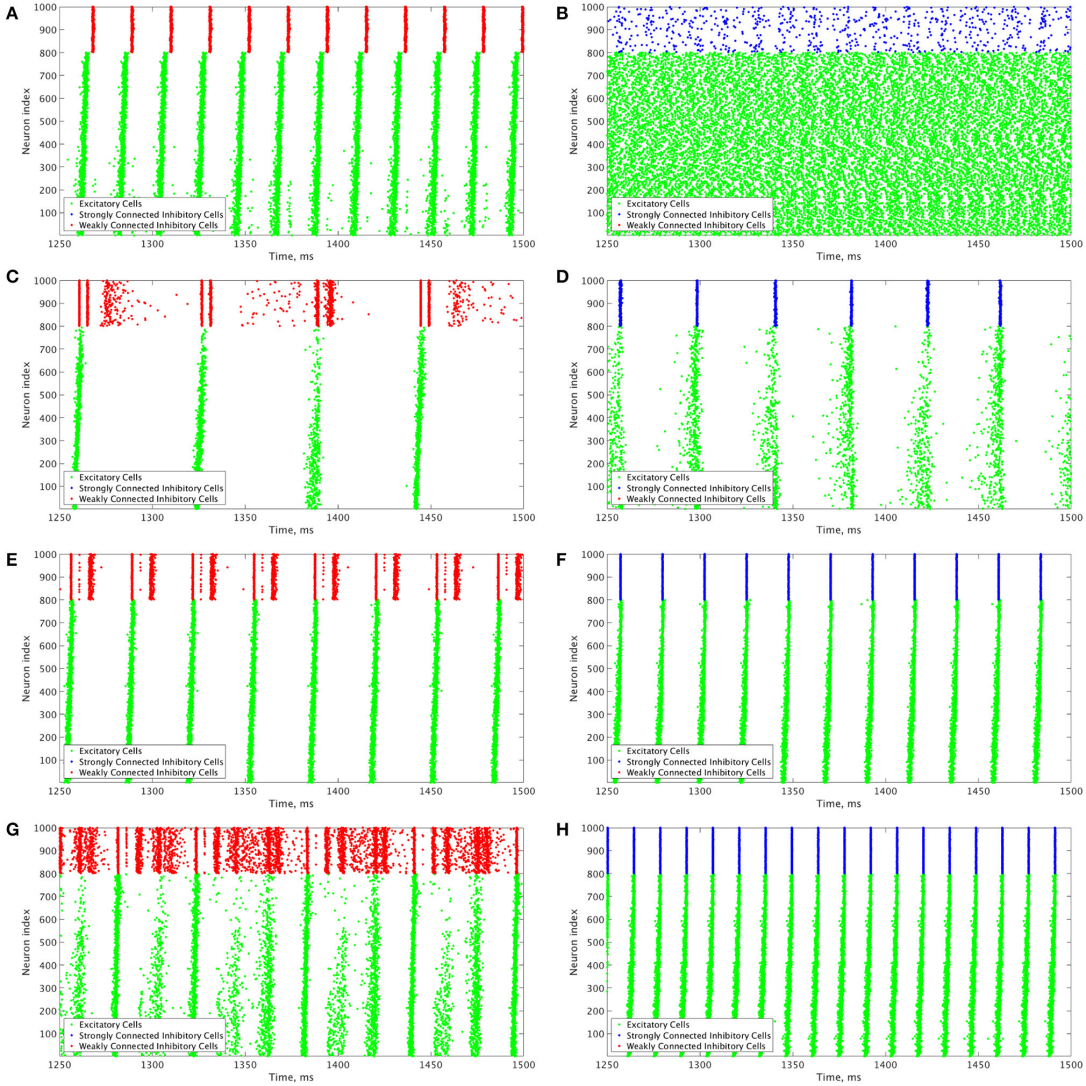
Example raster plots illustrate the differences between dynamics in networks with weakly connected and strongly connected inhibitory subnetworks. **(A–H)** Example raster plots with the excitatory cells (green dots) in these raster plots organized such that cells with the highest external drive are given the lowest Neuron Indices with the rest of the neurons organized such that decreasing external drive corresponds with increased Neuron Index. Panel letter corresponds with overlaid labels in Figures 4, 5 indicating the parameters of the given network. **(A,C,E,G)** are from networks with weakly connected inhibitory subnetworks, while **(B,D,F,H)** are from networks with strongly connected inhibitory networks. **(A,B)** are raster plots from a network with an E-I synaptic weight of 0.0004 mS/cm^2^ and an average intrinsic excitatory cell firing frequency of 98.8 Hz. **(C,D)** Are raster plots from a network with an E-I synaptic weight of 0.00235 mS/cm^2^ and an average intrinsic excitatory cell firing frequency of 39.6 Hz. **(E,F)** are raster plots from a network with an E-I synaptic weight of 0.00235 mS/cm^2^ and an average intrinsic excitatory cell firing frequency of 80 Hz. **(G,H)** are raster plots from a network with an E-I synaptic weight of 0.00235 mS/cm^2^ and an average intrinsic excitatory cell firing frequency of 126 Hz.

These results motivate the current study in which we describe how changing the I-I connectivity strength in an E-I network is the impetus behind changing pattern formation in the inhibitory network, which in turn affects the dynamics of the excitatory network. The PING dynamics that have been analyzed in the literature typically are analogous to those seen in Figures 3D,F,H, where the inhibitory network is strongly intra-connected and exhibits one instance of activity per oscillatory cycle. However, by weakening the I-I connectivity, different types of dynamics can arise amongst the inhibitory cells that affect the profile of excitatory network bursts, as seen by the examples in Figures 3C,E,G. We focus on the effects that multiple firings of inhibitory cells and the consistency and organization of these bursts have on the temporal organization of firing in excitatory cell bursts.

### E-I networks with strong and weak I-I synaptic strength

We analyze E-I networks with two I-I connectivity strengths in detail: an E-I network with strong intra-connectivity amongst the inhibitory subnetwork (entitled “Strong Networks” for brevity) and an E-I network with weak intra-connectivity amongst the inhibitory subnetwork (entitled “Weak Networks” for brevity). Strong Networks have an I-I synaptic weight of 0.025 mS/cm^2^, which was chosen in order to be analogous to the strong inhibitory synaptic weights used in our study of strictly inhibitory networks (Rich et al., 2016) (where scaling from 1,000 inhibitory neurons in the strictly inhibitory networks to 200 inhibitory neurons in the E-I networks is taken into account). Weak Networks have an I-I synaptic weight of 0.0015 mS/cm^2^.

For both Strong and Weak Networks we vary the average intrinsic excitatory cell firing frequencies and E-I synaptic weights. The I-E synaptic weight as well as connectivity densities (I-I, E-I, and I-E) are kept constant in all simulations.

**Figure 4:**
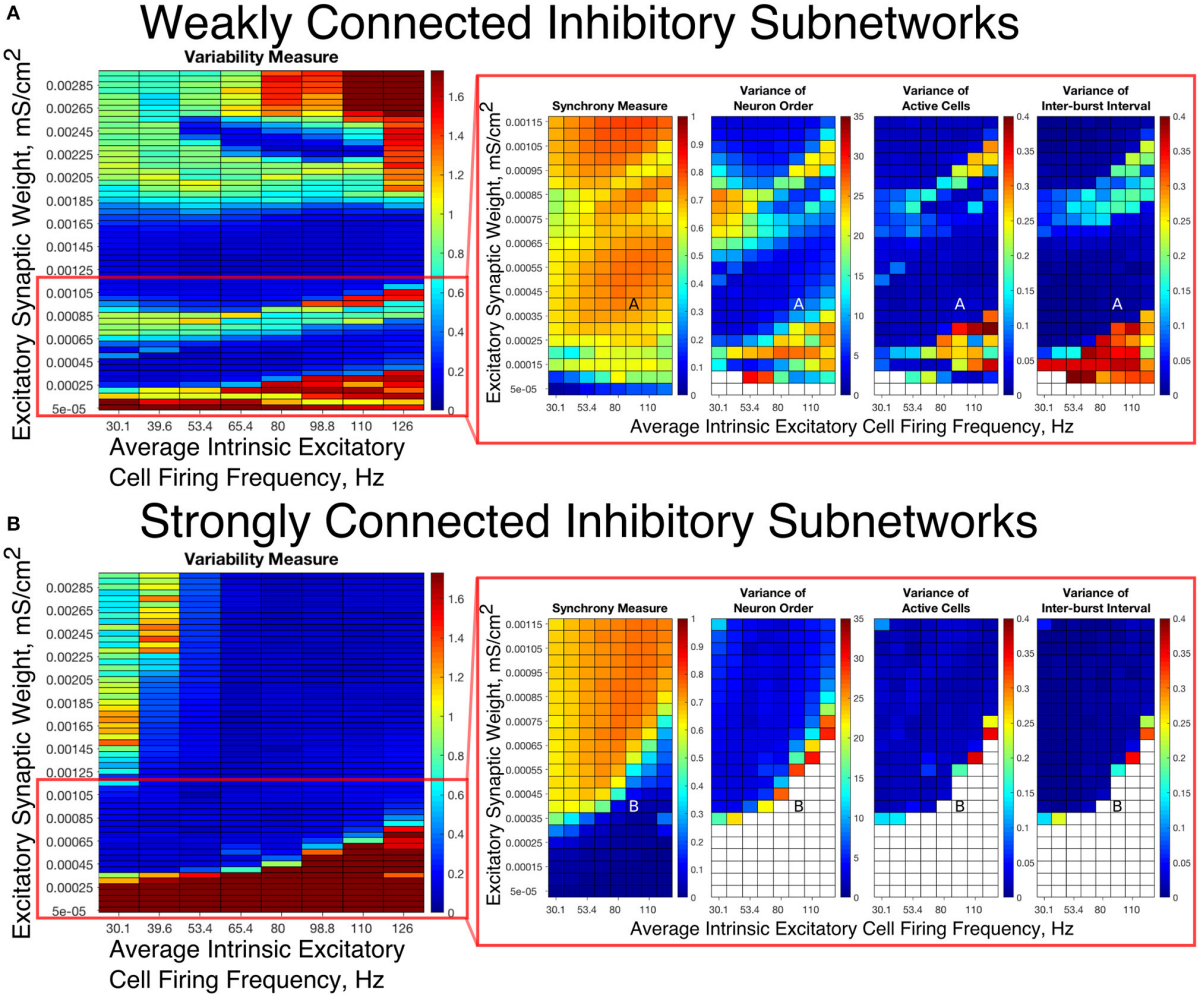
E-I networks with weakly connected inhibitory subnetworks are able to achieve synchrony for low values of the E-I synaptic weight, while E-I networks with strongly connected inhibitory subnetworks are unable to achieve any sort of excitatory bursting activity for many of these networks. **(A,B)** Variability Measure (left panel) calculated over the entire parameter range studied, with the parameter regime of particular interest outlined in red. For this parameter regime of interest, we show the Synchrony Measure along with the three measures that are used to calculate the Variability Measure (Variance of Neuron Order, Variance of Active Cells, and Variance of Inter-burst Intervals) in the red box making up the right panel. White entries in the heatmaps indicate that the excitatory network did not achieve sufficient synchrony for the given measure to be accurately calculated for that network. Overlaid letters indicate parameter values of example raster plots in Figure 3. Results for E-I networks with weakly connected inhibitory subnetworks are shown in **(A)**, while results for E-I networks with strongly connected inhibitory subnetworks are shown in **(B)**. In the parameter regime of interest, networks with weakly connected inhibitory subnetworks achieve synchrony of the excitatory subnetwork for many network parameters for which networks with strongly connected inhibitory subnetworks are completely asynchronous.

**Figure 5:**
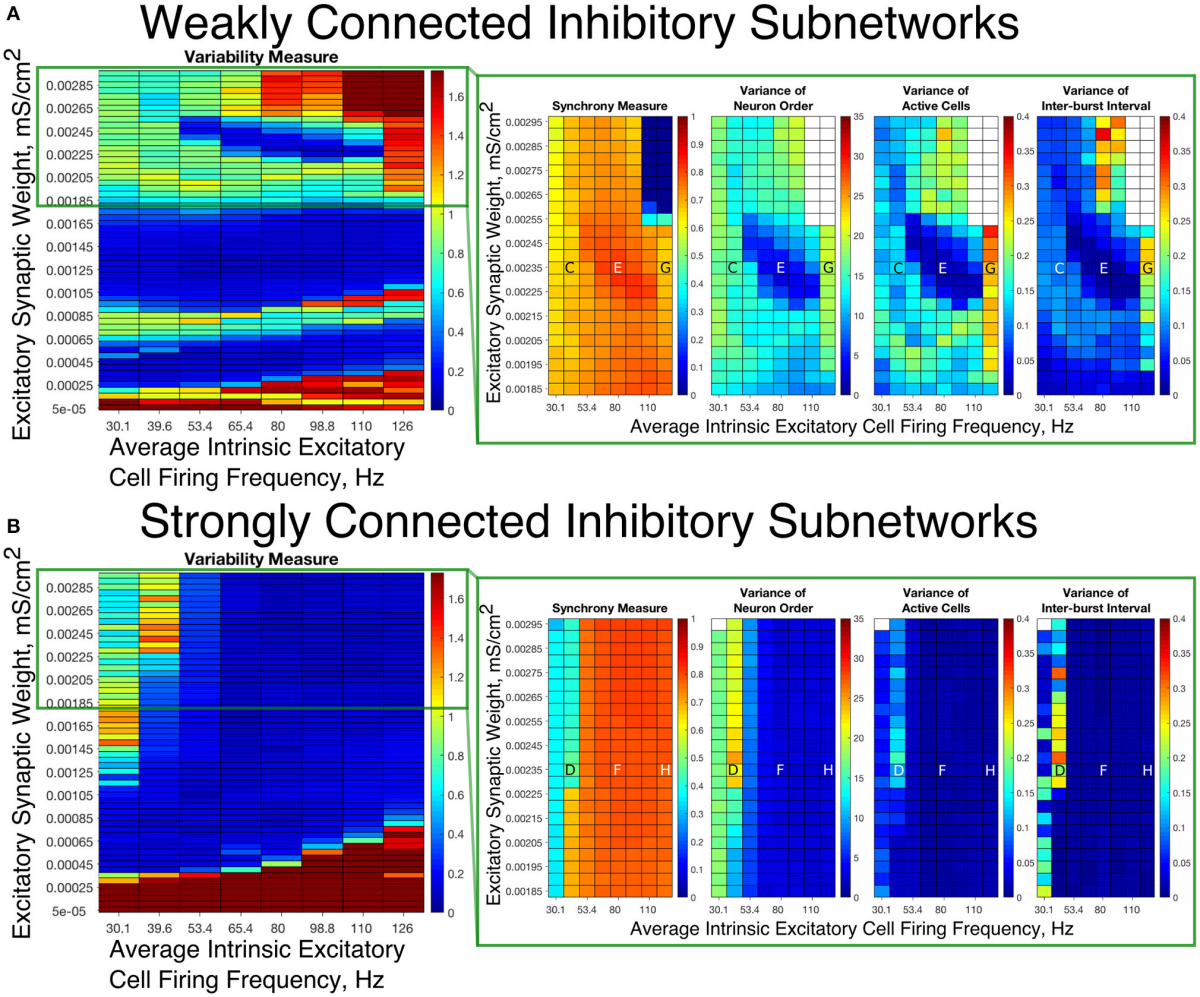
E-I networks with weakly connected inhibitory subnetworks exhibit excitatory bursting with high variability for high values of the E-I synaptic weight despite exhibiting synchrony in this parameter regime; in contrast, E-I networks with strongly connected inhibitory subnetworks exhibit mostly low variability firing in this parameter regime. **(A,B)** Variability Measure (left panel) calculated over the entire parameter range studied, with the parameter regime of particular interest outlined in green. For this parameter regime of interest, we show the Synchrony Measure along with the three measures that are used to calculate the Variability Measure (Variance of Neuron Order, Variance of Active Cells, and Variance of Inter-burst Intervals) in the green box making up the right panel. White entries in the heatmaps indicate that the excitatory network did not achieve sufficient synchrony for the given measure to be accurately calculated for that network. Overlaid letters indicate parameter values of example raster plots in Figure 3. Results for E-I networks with weakly connected inhibitory subnetworks are shown in **(A)**, while results for E-I networks with strongly connected inhibitory subnetworks are shown in **(B)**. In the parameter regime of interest, networks with strongly connected inhibitory subnetworks almost exclusively exhibit bursting patterns with low variability, while networks with weakly connected inhibitory subnetworks show a much higher Variability Measure due to the higher values of the Variance of Neuron Order, Variance of Active Cells, and Variance of Inter-burst Intervals for most networks in this parameter regime.

To summarize network dynamics, we show the Variability Measure for Strong Networks in the left panel of Figures 4A, 5A and for Weak Networks in the left panel of Figures 4B, 5B. For moderate values of the E-I synaptic weight, both Strong and Weak networks show synchronous rhythmic bursting with low Variability Measure reflecting periodic, well-organized excitatory cell bursts. For low E-I synaptic weight and high E-I synaptic weight there is a significant difference that requires further investigation. The right panels of Figures 4A,B investigate networks with low E-I synaptic weight in more detail by showing the Synchrony Measure as well as the three measures that are used in the formation of the Variability Measure individually. The same is done for networks with high E-I synaptic weight in the right panels of Figures 5A,B.

Our results indicate that Strong Networks do not display any form of excitatory bursting activity in most networks with our lowest E-I synaptic weights. This is illustrated by the Synchrony Measure in the right panel of Figure 4B. A raster plot showing an example of this asynchronous activity is shown in Figure 3B. In contrast, Weak Networks achieve excitatory bursting for many networks with our lowest E-I synaptic weights as shown by the Synchrony Measure in the right panel of Figure 4A. The raster plot in Figure 3A shows a network with identical parameters to that shown in Figure 3B with only the I-I synaptic weight weakened which exhibits clear bursting activity. Additionally, there is a consistent ordering of the excitatory cells in each burst, along with periodic firing and the same level of participation of excitatory cells in each burst, leading to low values of the Variability Measure.

When E-I synaptic weight is strengthened, strong inhibitory intra-connectivity plays a significant role in controlling network dynamics. For a large majority of the networks with high E-I synaptic weight (highlighted in Figure 5B), Strong Networks show well-organized synchronous bursting with very low Variability Measures, with behavior typified by the example raster plots shown in Figures 3F,H. In contrast, a majority of Weak Networks show a significantly increased Variability Measure in the regime of high E-I synaptic weight, despite still exhibiting synchronous excitatory activity. As shown in the right panel of Figure 5A, these networks show noticeable increases in each of the measures making up the Variability Measure. High values of the Variance of Neuron Order indicates that the timing of individual excitatory neuron activity within each burst is not consistent (shown by the “wider” excitatory bursts without a clear slope and outlier firings in the raster plots displayed in Figures 3C,G); high values of the Variance of Active Cells indicates that the number of excitatory cells in each excitatory burst fluctuates significantly from burst to burst (again illustrated by Figures 3C,G); increased values of the Variance of Inter-burst Interval indicates that excitatory burst firing is not strictly periodic (best illustrated by Figure 3G). All three of these issues, reflected by an increase in the Variability Measure, imply that for high E-I coupling, Weak Networks lose the organization, consistency and strict periodicity of excitatory bursts that have classically typified PING rhythmicity.

The cause of the changes in excitatory bursting dynamics in our Weak Networks is the disorganization of inhibitory cell firing. As illustrated by the example raster plots in Figures 3C,G, the combination of weak I-I and strong E-I synaptic strength leads to multiple instances of inhibitory cell activity in response to a burst of excitatory cell activity. Due to the randomness in network connectivity and cell heterogeneity, these multiple bursts are not consistent across different instances of inhibitory activity; in extreme cases, inhibitory bursts may not exhibit clear synchrony. The specific form of the inhibitory network activity changes throughout the simulation, altering in turn the modulation of the excitatory network's activity. Thus, while these dynamics might not disrupt the formation of synchronous excitatory bursts, they do disrupt the organization, consistency and periodicity of these bursts.

However, there is a parameter regime within the highlighted high E-I synaptic strength region for which Weak Networks retain a low Variability Measure. Such networks, an example of which is shown by the raster in Figure 3E, still exhibit multiple bursts of inhibitory activity in response to excitatory activity, but do so in a consistent and organized fashion in response to each instance of excitatory activity. The existence of such networks shows that it is the disorganization of inhibitory cell firing, and not necessarily the existence of multiple inhibitory network bursts, that causes significant changes to the properties of excitatory bursts.

An additional parameter regime of interest is networks with a low average intrinsic cell firing frequency but a high E-I synaptic weight. Here, Strong Networks exhibit a significantly increased Variability Measure similarly to Weak Networks, albeit driven by a different mechanism. While the increased variability in Weak Networks in this regime can be attributed to overactivity and disorganization amongst the inhibitory cells (as illustrated by the raster in Figure 3C), the increased variability in this regime for Strong Networks is caused primarily by the dynamics of the excitatory cells.

A combination of two factors leads to these dynamics in Strong Networks, typified by the behavior shown by the raster plot in Figure 3D. First, for networks with the slowest average intrinsic excitatory cell firing frequencies, following an inhibitory burst, the excitatory cells are slow to fire leading to a longer interval between inhibitory bursts. This allows the burst of excitatory activity to occur over a longer period of time (see example in Figure 3D). Thus, the possibility of more variability in the excitatory bursts arises. In contrast, for networks with faster firing excitatory cells, the excitatory cells fire shortly after the inhibition decays, causing the next inhibitory burst to occur quickly as well. This, in turn, creates a very small time window in which excitatory activity can occur and thus less possibility for significant variability in firing times.

Second, with high E-I synaptic weight, even strongly intra-connected inhibitory networks can receive sufficient excitatory signal to burst without a majority of the excitatory cells firing. Excitatory cells that fire before the burst of inhibition are suppressed following the burst of inhibition, while cells that have not fired are typically past the threshold for action potential firing at the time of the burst of inhibition, and thus their firing pattern is not significantly affected by the inhibition. Thus, cells that fire prior to the burst of inhibition on one cycle will fire later, if at all, on the following cycle. This causes disorganization in the neuron order from cycle to cycle, as the driving current to the excitatory cells is now not the only factor determining when in a burst they fire, since each neuron is not receiving similar inhibitory delay as is typical in PING rhythmicity.

These two factors cooperatively lead to the disorganization of the excitatory bursts as reported by the Variability Measure. This is shown in the raster in Figure 3D by the lack of a clear slope in the excitatory burst, as well as the bursts occurring over a longer timespan. Additionally, the number of cells participating in each burst varies significantly by the same reasoning.

In summary, in most cases high values of the E-I synaptic weight require stronger inhibitory intra-connectivity in order to preserve consistent inhibitory response to excitatory activity in an E-I network, which in turn preserves well-ordered and consistent bursting of the excitatory population. However, our investigation into these networks also reveals that Weak Networks may exhibit well-organized and consistent excitatory bursting when the inhibitory network exhibits multiple bursts, as long as such bursts are themselves well-organized. Additionally, Strong Networks exhibit increased variability for high E-I synaptic weight when intrinsic excitatory cell firing has lower average frequency.

**Figure 6:**
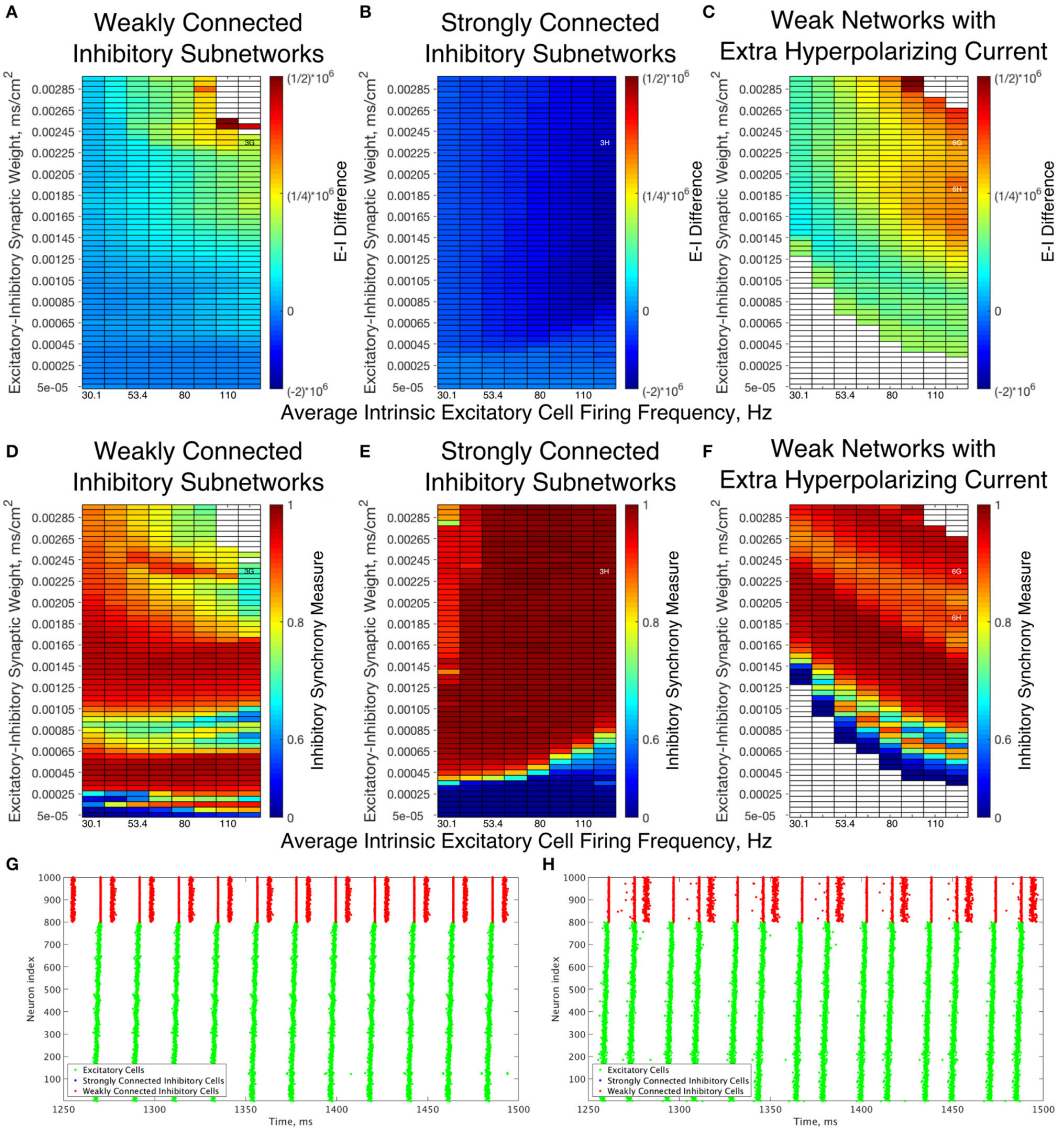
Dynamical differences between networks with weakly connected and strongly connected inhibitory subnetworks are reflected in differences in E-I Difference and Inhibitory Synchrony Measure, even with changes to the external hyperpolarizing current. **(A–C)** Synaptic E-I Difference for Weak Networks **(A)**, Strong Networks **(B)**, and Weak Networks where the external hyperpolarizing current is increased from −0.2 to −3.0 μA/cm^2^ **(C)**. **(D–F)** Inhibitory Synchrony Measure for the same three network types. White entries in the heatmaps indicate that the measure could not be calculated due to insufficient inhibitory activity, and overlaid alphanumeric codes indicate position of example raster plots seen in Figure 3 (for comparison to those shown here) and in this figure. **(G,H)** Example raster plots for Weak Networks with Extra Hyperpolarizing Current; both examples are for a network with an average intrinsic excitatory cell firing frequency of 126 Hz, with **(G)** an example from a network with an E-I synaptic weight of 0.00235 mS/cm^2^ while **(H)** is an example from a network with an E-I synaptic weight of 0.00190 mS/cm^2^. Weak Networks, both with and without additional hyperpolarizing current, show a dominance of excitatory synaptic activity reflected in positive E-I Difference values that increase as the E-I synaptic weight increases, while Strong Networks show a dominance of the inhibitory synaptic activity reflected in largely uniform negative values of the E-I Difference. Moreover, Weak Networks with Extra Hyperpolarizing Current retain distinct behaviors from Strong Networks as illustrated by the Inhibitory Synchrony Measure and example raster plots.

A further explanation as to the differences underlying the Strong and Weak Networks lies in the synaptic E-I Difference in the inhibitory subnetwork. As shown in Figure 6, there is a stark difference between the E-I Difference for Weak Networks (Figure 6A) and Strong Networks (Figure 6B). In particular, the E-I Difference for Strong Networks is always negative, meaning that the inhibitory intra-connectivity dominates the excitatory drive to the inhibitory cells, and shows minimal change in response to altering network parameters. In contrast, the E-I Difference for Weak Networks increases as the E-I synaptic weight increases and is always positive, meaning that the excitatory drive dominates the inhibitory intra-connectivity within the inhibitory subnetwork.

This dichotomy provides a more quantitative explanation for the dynamical differences exhibited by these networks. The dominance of inhibitory intra-connectivity over the excitatory synaptic drive in Strong Networks ensures that following a burst of inhibitory activity, the inhibitory synaptic drive dominates the excitatory synaptic drive and ensures that inhibitory cells are silent following the burst. This explains the tendency for Strong Networks to only exhibit the 1:1 bursting ratio that is a hallmark of classic PING theory, as well as the minimal differences in network dynamics seen as the E-I synaptic weight increases. However, in Weak Networks, increasing E-I synaptic weight cannot be counteracted by the weaker inhibitory synapses as illustrated by the increasing and positive E-I Difference, which allows for multiple bursts of inhibitory activity that are often disorganized and in turn lead to an increase in the Variability Measure.

Additionally, analyzing the E-I Difference cements the importance of the I-I connectivity in dictating overall network dynamics. Figure 6C illustrates the E-I Difference for a Weak Network where the hyperpolarizing current to the inhibitory cells is increased from −0.2 to −3.0 μA/cm^2^. In this network paradigm, the inhibitory cells are “less excitable” than those in the Weak Networks due to the external current, but the synaptic E-I Difference in the inhibitory subnetwork retains similarity to that seen in the Weak Network, namely retaining positive values that increase with increasing E-I synaptic weight. Importantly, the E-I Difference remains entirely distinct from that of Strong Networks, which can be considered to have “less excitable” inhibitory cells given the stronger I-I connectivity. This result indicates that the net excitability of the inhibitory cells and the strength of inhibitory intra-connectivity are distinct features that have differing effects on network dynamics. Indeed, multiple and sometimes disorganized inhibitory bursts are seen in Weak Networks with this additional hyperpolarizing current, as shown by the example raster plots in Figures 6G,H, while Strong Networks never show inhibitory double bursts. This again shows that making the cells less excitable through an external hyperpolarizing current does not have the same effect as doing so by increasing the I-I synaptic weight. The differences in dynamics of the inhibitory cells between these networks and Strong Networks are confirmed by comparing the Synchrony Measure computed for the inhibitory subnetwork, shown for all three types of networks discussed above in Figures 6D–F. Thus, regardless of the hyperpolarizing current to the inhibitory cells, the weak I-I synaptic weight is not sufficient to balance increasing excitatory signal, preventing them from achieving the very synchronous bursts exhibited in Strong Networks for all values of the E-I synaptic weight.

**Figure 7:**
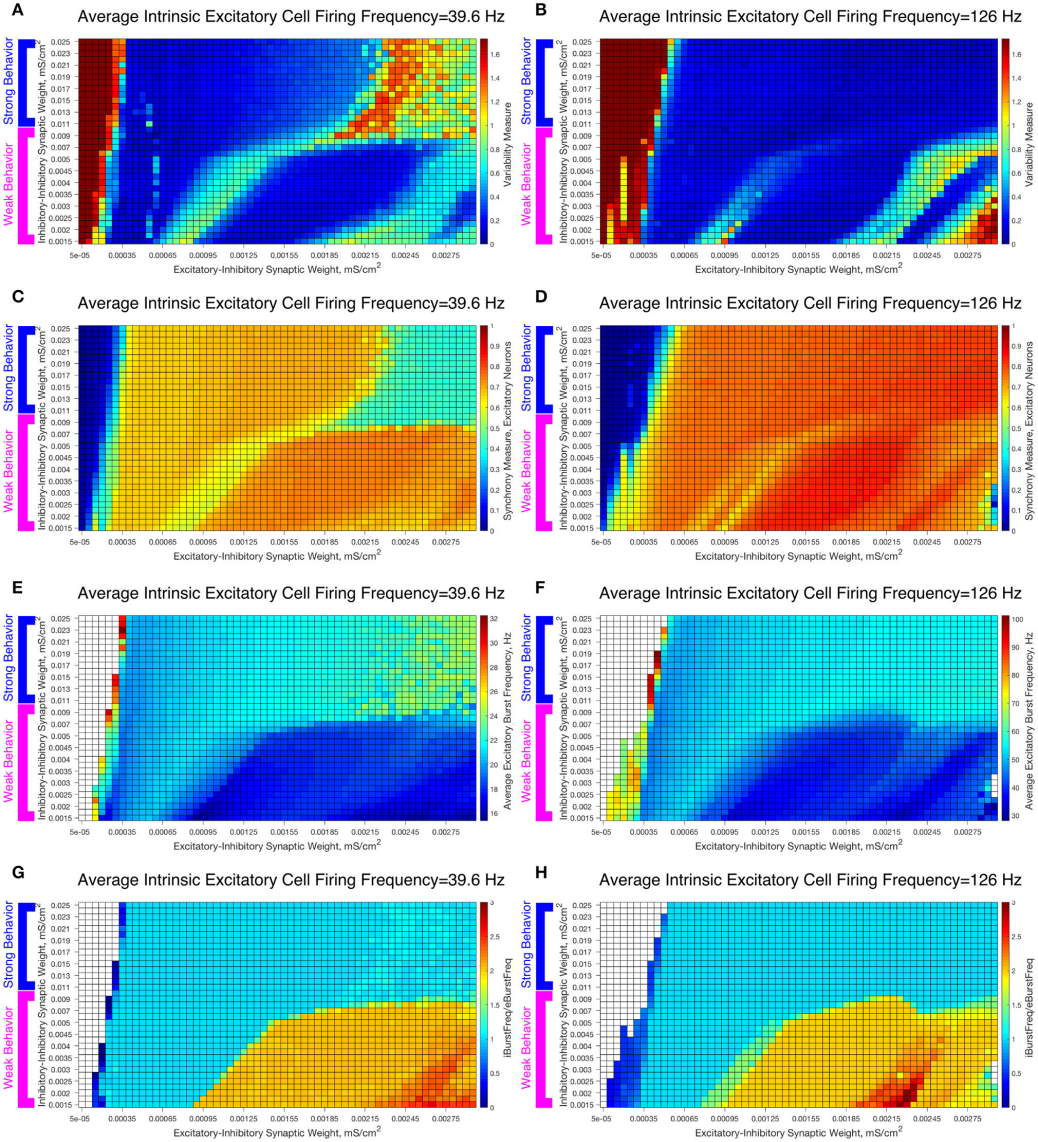
Varying the I-I synaptic weight reveals that E-I networks display two distinct dynamical patterns directly analagous to those seen in our networks with weakly connected or strongly connected inhibitory subnetworks. **(A–H)** Heatmaps varying the E-I synaptic weight on the x-axis and I-I synaptic weight on the y-axis for networks with an average intrinsic excitatory cell firing frequency of 39.6 Hz **(A,C,E,G)** and 126 Hz **(B,D,F,H)**. Four measures are shown: the Variability Measure **(A,B)**, the Synchrony Measure for Excitatory Neurons **(C,D)**, the Average Excitatory Burst Frequency in Hz **(E,F)** and the ratio of the Average Inhibitory Burst Frequency over the Average Excitatory Burst Frequency **(G,H)**. White boxes in the heatmaps indicate that the excitatory network did not achieve sufficient synchrony for the given measure to be accurately calculated for that network. Values of I-I connectivity strength that exhibit behavior corresponding with that seen in our network with a weakly connected inhibitory subnetwork are highlighted by the pink bracket, while values that exhibit behavior corresponding with that seen in our network with a strongly connected inhibitory subnetwork are highlighted by the blue bracket.

To study the robustness of these behaviors, we varied the I-I synaptic weights between the values used in the Weak and Strong Networks. The results are illustrated in Figure 7, in which we vary the I-I synaptic strength along the y-axis and the E-I synaptic strength along the x-axis while keeping the average intrinsic cell firing frequency fixed. We show examples for slow firing excitatory cells in Figures 7A,C,E,F and for fast firing excitatory cells in Figures 7B,D,F,G.

As the I-I connectivity is varied, there are two distinct dynamical regimes, one with dynamics analogous to the Strong Network (blue bracketed values) and one with dynamics analogous to the Weak Network (pink bracketed values), with an abrupt transition between the two. This result justifies our study of the Strong Network and Weak Network as their activity is representative of dynamics for a range of I-I synaptic weights in our E-I network topology. For the values of the I-I synaptic weight in the weak regime, the Variability Measure (Figures 7A,B) increases non-monotonically as E-I synaptic weight increases as discussed for the Weak Network. These regimes of high and low Variability Measure shift as the I-I synaptic weight increases since the E-I synaptic weight for which a given behavior is achieved likewise increases; for example, when the I-I synaptic weight is increased, a correspondingly higher E-I synaptic weight, which provides a stronger drive to the inhibitory cells, is required to achieve the “parameter balance” necessary for these networks to achieve a low Variability Measure despite a high E-I synaptic weight. Additionally, these networks display a non-zero Synchrony Measure (Figures 7C,D) for lower values of the E-I synaptic weight. In contrast, the values of the I-I synaptic weight in the strong regime display a consistently low Variability Measure (with the exception of high E-I synaptic weight for the slower firing network, the unique situation discussed in detail above) but completely asynchronous activity (shown by the Synchrony Measure) for low values of the E-I synaptic weight.

The presence of two distinct dynamical regimes is also apparent from analyzing the average excitatory burst frequency (Figures 7E,F) and the ratio of inhibitory bursts to excitatory bursts (Figures 7G,H) in these networks, properties that reveal the network dynamics in more detail. Networks with inhibitory intra-connectivity in the strong regime show a monotonic increase in their average excitatory burst frequency as the E-I synaptic weight increases, while networks with inhibitory intra-connectivity in the weak regime show an overall decrease in their average excitatory burst frequency as the E-I synaptic weight increases, with some instances of increasing burst frequency that correspond with networks that exhibit low variability. Additionally, networks in the strong regime exclusively exhibit a 1:1 ratio between inhibitory and excitatory bursts, while networks in the weak regime can exhibit two or even three inhibitory bursts for each excitatory burst. Through these measures we see that activity closely matching classic PING rhythms is seen over the majority of the values of the E-I synaptic weight for the range of I-I synaptic weights that yield behavior analogous to the Strong Network studied above, while unique dynamics are seen over the range of I-I synaptic weights that yield behavior analogous to the Weak Network studied above.

These results reveal the robustness of the dichotomous dynamics displayed by excitatory cells in the Strong Network and Weak Network studied above. Indeed, it appears that slight heterogeneities in the I-I synaptic strength should not lead to major changes in network dynamics in an E-I network, while larger heterogeneities (where I-I synaptic weights include those inducing both weak and strong behavior) may lead to antithetical dynamics. This motivates our construction of E-I networks with heterogeneous inhibitory populations, described below.

### E-I networks with noisy excitatory cells

To confirm the robustness of our results to more realistic biological conditions, we simulated analogous networks while adding Poisson trains of excitatory synaptic input to the excitatory neurons. These simulations were performed with a range of noise amplitudes: the lowest amplitude noise slightly accelerates the next firing of the perturbed neuron, while the highest amplitude noise causes the perturbed neuron to fire with a probability of nearly 1 in a 5 ms window following the perturbation.

**Figure 8:**
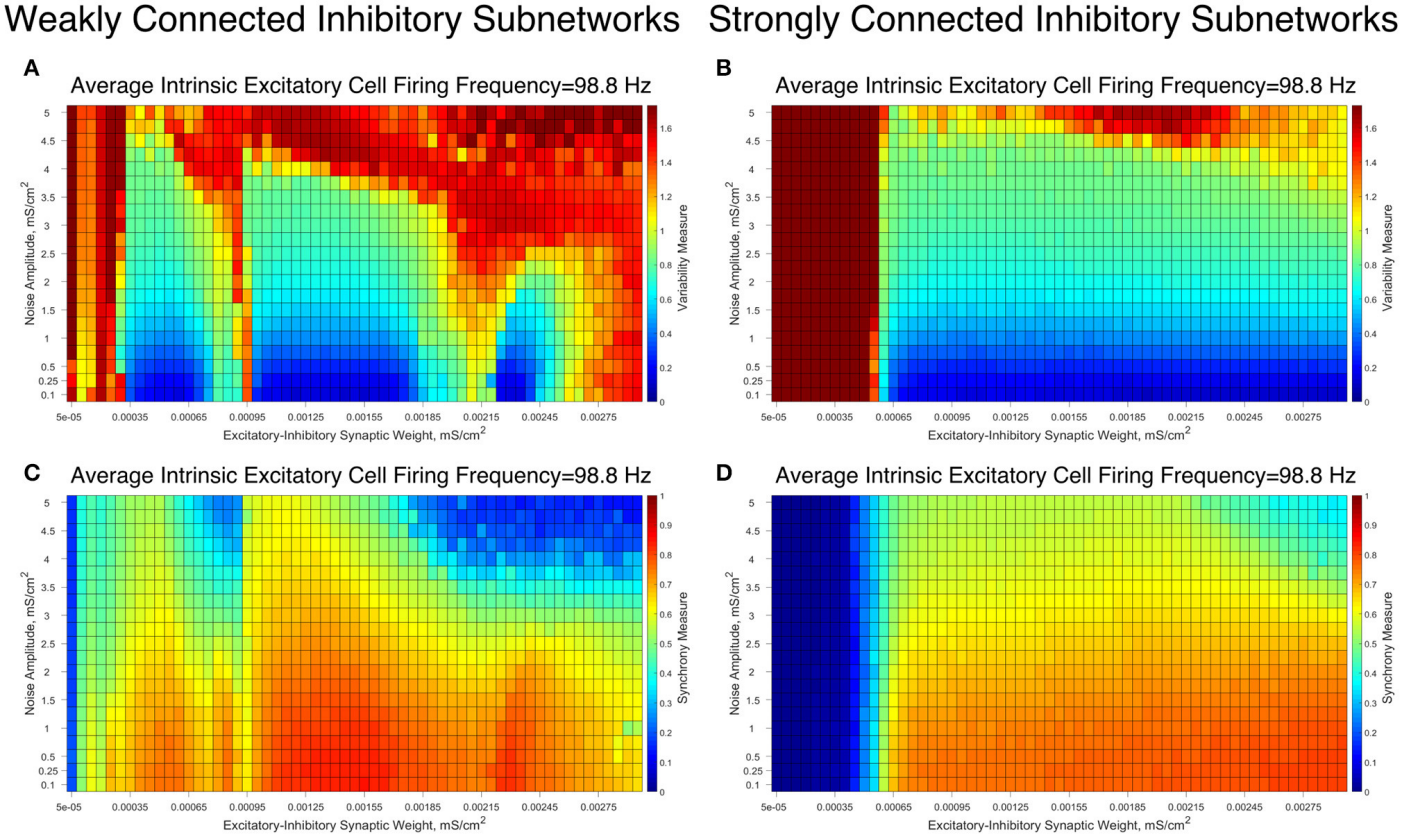
Differences in dynamical patterns between Weak and Strong Networks are preserved in the presence of noisy drive to the excitatory cells. **(A–D)** Variability Measure **(A,B)** and Synchrony Measure **(C,D)** shown for Weak Networks **(A,C)** and Strong Networks **(B,D)** with an average intrinsic excitatory cell firing frequency of 98.8 Hz in the presence of noise with varying amplitudes (y-axis). E-I synaptic weight is varied along the x-axis. Distinct differences in the dynamics articulated by the Variability Measure and Synchrony Measure are still seen between Weak and Strong Networks, with the major similarity being that both networks similarly devolve into asynchronous excitatory cell firing with high-amplitude noise.

The results of these simulations for an illustrative choice of the average intrinsic excitatory cell firing frequency, for both Strong Networks and Weak Networks, are illustrated in Figure 8. For both types of networks, the network dynamics are quantified via the Variability Measure (Figures 8A,B) and Synchrony Measure (Figures 8C,D). Overall, we observe that slight increases in the Variability Measure are seen as the noise amplitude increases, but this increase is largely uniform across all values of the E-I synaptic weight, preserving the relative pattern of well-organized and less organized excitatory bursting dynamics. These patterns only break down in the presence of large amplitude noise, in which many simulations lose synchronous excitatory activity, shown by the maximal Variability Measure values and near-minimal Synchrony Measure values.

In particular, Weak Networks (Figures 8A,C) still exhibit pockets of lower Variability Measure amidst the simulations with higher E-I synaptic strength that tend to exhibit higher Variability Measure. In contrast, Strong Networks (Figures 8B,D) retain their more consistent pattern of exhibiting low Variability Measure in nearly every case where excitatory synchrony is achieved. In both scenarios, the Variability Measure increases in a largely consistent manner as the amplitude of the noise increases, with exceptions for the scenarios when excitatory network synchrony is completely lost.

These results taken together illustrate that the introduction of noise does not significantly alter the previously identified dynamical regimes of the Strong and Weak Networks, as each retains their unique properties that differentiate network dynamics dependent upon the strength of inhibitory intra-connectivity.

### E-I networks with heterogeneity in I-I synaptic strength

**Figure 9:**
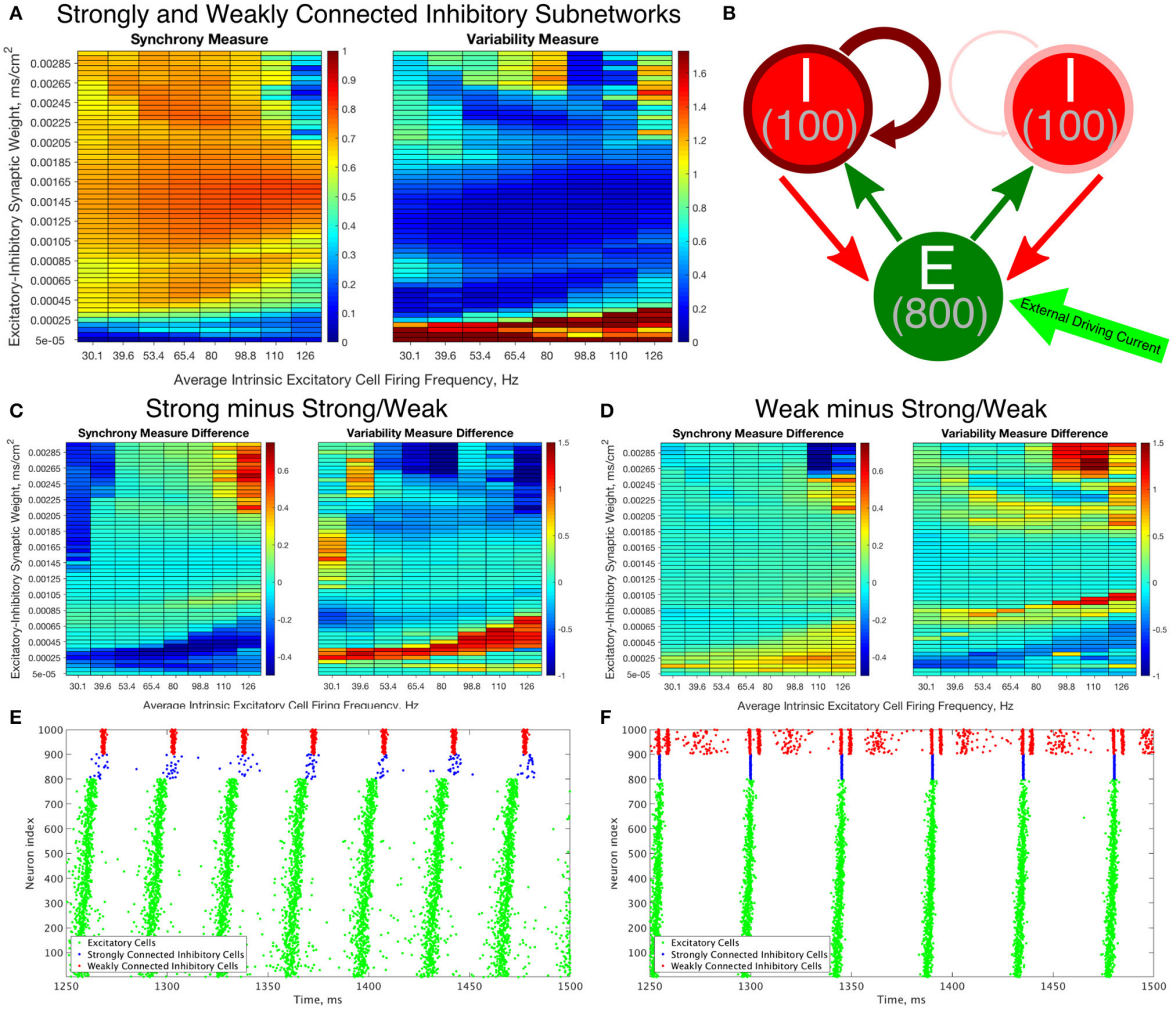
Constructing an E-I network that contains both strongly connected and weakly connected inhibitory subnetworks decreases burst variability in networks with strictly weakly connected inhibitory subnetworks while also expanding the parameter regime in which any synchrony is achieved in comparison to networks with strictly strongly connected inhibitory subnetworks. **(A)** Synchrony Measure (left) and Variability Measure (right) for a network with both strongly connected and weakly connected inhibitory subnetworks (hereafter referred to as Strong/Weak networks). **(B)** Diagram representing the connectivity for our Strong/Weak network. The thicker and darker red curve connecting the inhibitory cells to themselves for the population on the left illustrates the strong interconnectivity of those interneurons, while the thinner and lighter red curve connecting the inhibitory cells to themselves for the population on the right illustrates the weak interconnectivity of those interneurons. **(C,D)** Difference between the Synchrony and Variability Measure of a strictly weakly connected inhibitory subnetwork **(C)** and strictly strongly connected inhibitory subnetwork **(D)** with our Strong/Weak network with networks show the parameter regimes in which the Strong/Weak Networks show a higher Synchrony Measure and lower Variability Measure compared to networks with only one strength of inhibitory interconnectivity. **(E,F)** Example raster plots from Strong/Weak Networks. **(E)** is a network with an intrinsic cell firing frequency 53.4 Hz and an E-I synaptic weight of 0.0003 mS/cm^2^, and is from a parameter regime similar to the network shown in Figure 5B. **(F)** is a network with an intrinsic cell firing frequency 53.4 Hz and an E-I synaptic weight of 0.00225 mS/cm^2^, and is from a parameter regime similar to the network shown in Figure 5C.

To construct a network that incorporates properties of networks with weak I-I connectivity and networks with strong I-I connectivity, we created networks with heterogeneous inhibitory synaptic strengths (which for brevity we will refer to as Strong/Weak Networks, Figure 9B).

The Strong/Weak Network contains 800 excitatory cells and 200 inhibitory cells as before, but the inhibitory cells are divided into two subnetworks of 100 cells each. One of the subnetworks has a strong I-I synaptic strength of 0.05 mS/cm^2^, while the other has a weak I-I synaptic strength of 0.003 mS/cm^2^. The values of the I-I synaptic strength are scaled from the values used in the Strong Networks and Weak Networks for a network of 100 as opposed to 200 cells. Each of these subnetworks has 30% intra-connectivity density, just as for the inhibitory subnetworks in our previously studied E-I networks, but these inhibitory neurons only synapse onto other inhibitory neurons within their subnetwork. Interneurons have the same likelihood of receiving synaptic input from an excitatory cell or sending synaptic output to an excitatory cell as in our networks studied above.

The Synchrony and Variability Measures for the excitatory neurons in Strong/Weak Networks are shown in Figure 9A. For ease of comparison the difference between these measures in the Weak and Strong Networks (Figures 4, 5) and the Strong/Weak Networks are shown in Figures 9C,D. As Figure 9C illustrates, compared to the Strong Networks, Strong/Weak Networks achieve a higher Synchrony Measure and lower Variability Measure for low values of the E-I synaptic weight. A raster plot exhibiting such a network is shown in Figure 9E, where despite sparse, asynchronous activity of the strongly connected interneurons, the weakly connected interneurons exhibit synchronous bursting that provides the necessary inhibition to the excitatory cells to promote synchronous bursting. In contrast, Strong Networks in this parameter regime are typified by the behavior shown by the raster in Figure 3B, which is completely asynchronous. Additionally, Strong/Weak networks exhibit higher values of the Synchrony Measure and lower values of the Variability Measure in the regime of high E-I synaptic weight and low average intrinsic cell firing frequency for which Strong Networks show less organized bursting.

Furthermore, as illustrated in Figure 9D, compared to Weak Networks, Strong/Weak Networks show a significant decrease in the Variability Measure for high values of the E-I synaptic weight, as well as in a thin parameter regime with moderate E-I synaptic weight for which Weak Networks showed increased variability in excitatory bursting. Figure 9F displays an example raster plot of a Strong/Weak network with high E-I synaptic weight where the organization and consistency of excitatory bursting is largely maintained thanks to consistent synchronous bursting from the strongly connected inhibitory neurons, which in turn helps to maintain a more consistent firing pattern amongst the weakly connected inhibitory neurons by gating excitatory cell activity. This can be compared to the Weak Network illustrated in Figure 3C which displays distinctly unorganized and variable bursting patterns.

Indeed, Strong/Weak Networks achieve the proverbial “best of both worlds,” exhibiting synchrony for very low values of the E-I synaptic weight like Weak Networks while preserving the organization and consistency of excitatory bursting for high values of the E-I synaptic weight like Strong Networks. This new type of E-I network with heterogeneity amongst the inhibitory interneurons thusly generates well-organized and consistent excitatory bursting over a wider parameter range than a network with homogeneous inhibitory intraconnectivity, regardless of the strength of that intraconnectivity. We will discuss the biological motivations for creating such a network and the implications of this mechanism in more detail in the Discussion below.

### E-I networks with type II interneurons

Our previous work revealed that intrinsic cellular properties, typified by the Type I and Type II neuron classifications, play a pivotal role in determining network dynamics in strictly inhibitory neural networks (Rich et al., 2016). The importance of interneuron cell type in those networks begs the question of whether E-I networks with different inhibitory cell types will exhibit different responses to a change in the I-I synaptic weight.

To probe this topic, we study E-I networks with the same topology as those studied above while replacing our Type I interneuron with either a model neuron exhibiting Type II properties without spike frequency adaptation (hereafter simply referred to as Type II neurons) or Type II properties with spike frequency adaptation (hereafter simply referred to as Type II neurons with adaptation).

**Figure 10:**
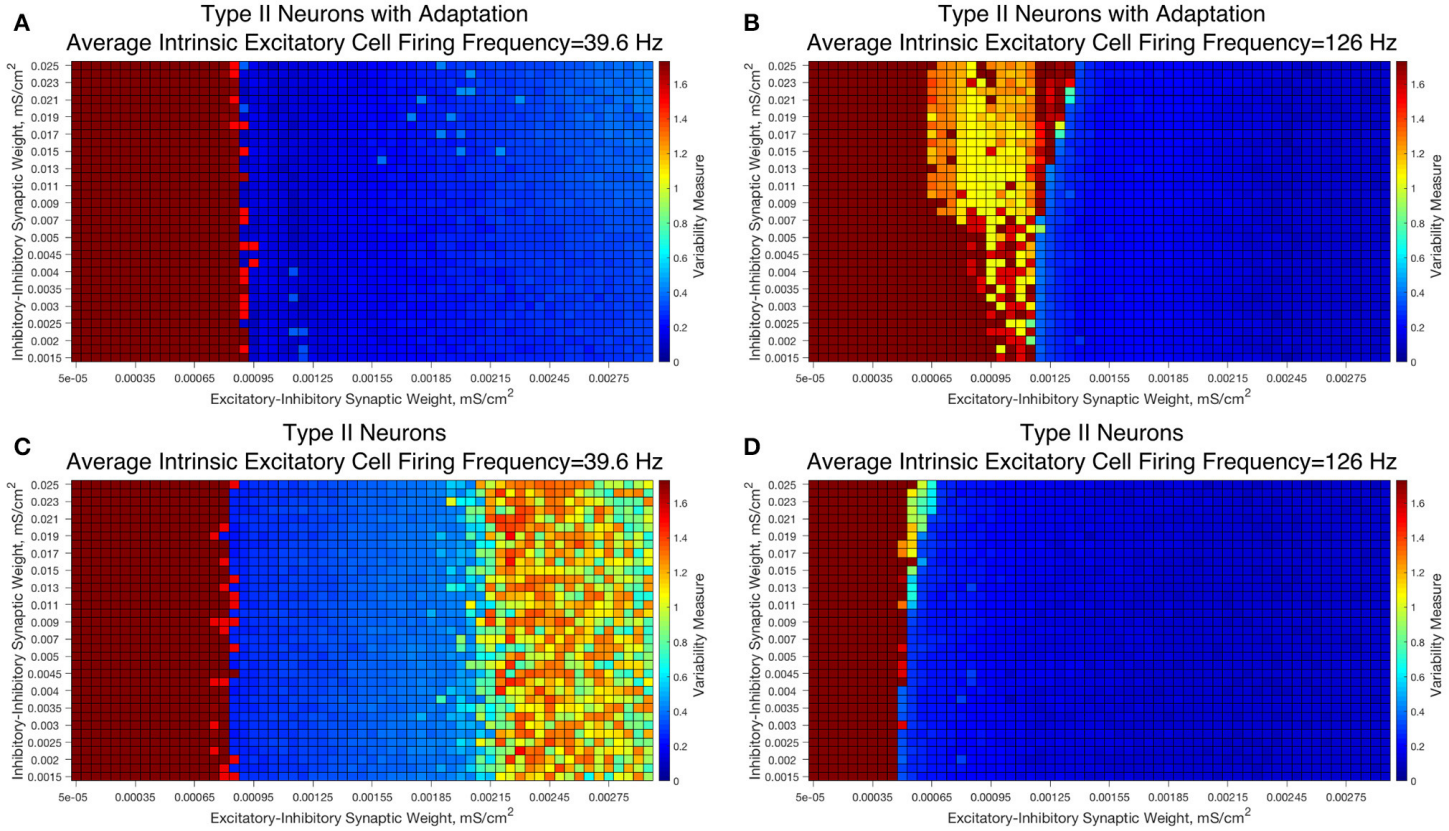
E-I networks with Type II interneurons, both with and without an adaptation current, do not show significant change in dynamics as a function of I-I synaptic strength, unlike E-I networks with Type I interneurons. **(A–D)** Heatmaps showing the Variability Measure for networks with varying E-I synaptic strength on the x-axis and varying I-I synaptic strength on the y-axis. The average intrinsic cell firing frequency of the networks are set at 39.6 Hz in **(A,C)** and 126 Hz in **(B,D)**. Results with inhibitory neurons modeled as a Type II neuron with adaptation are shown in **(A-B)**, while results with inhibitory neurons modeled as a Type II neuron without adaptation are shown in **(C,D)**. Neither networks with Type II with adaptation or Type II interneurons show the significant changes in dynamics as a function of the I-I synaptic weight that typified networks with Type I interneurons.

We observed that networks with either Type II interneuron do not exhibit significant changes in dynamics as the I-I synaptic weight changes. For slow firing networks there is essentially no change in the excitatory bursting properties as the I-I synaptic weight changes, as displayed in Figures 10A,C; indeed, even though networks with Type II neurons show a significantly increased Variability Measure for high values of the E-I synaptic weight, this increase is not dependent upon the I-I synaptic weight.

Faster firing networks with Type II neurons, shown in Figures 10B,D, also exhibit minimal change in network dynamics as a result of changing I-I synaptic weight. For the faster firing networks with Type II neurons with adaptation shown in Figure 10B, networks with high values of the I-I synaptic weight exhibit a regime with a moderate value of the Variability Measure whereas networks with weaker values of the I-I synaptic weight are less likely to do so; however, all networks, regardless of I-I synaptic strength, with moderate to high E-I synaptic weight still show similar bursting properties, unlike in our Type I networks.

These results imply that classic PING rhythmic bursting, which typically yield low values of the Variability Measure, are more robust to changes in I-I synaptic weight when the interneurons are Type II (with or without adaptation) then when the interneurons are Type I. This provides further evidence for the important role that cell type plays in networks with inhibitory neurons. Furthermore, these results also match the intuition we gained from varying the connectivity density in strictly inhibitory networks with these types of neurons, as unlike networks of Type I neurons, networks of Type II neurons showed little if any change in dynamics as the synaptic strength changed (Rich et al., 2016).

## Discussion

Our work here has shown that the strength of intra-connectivity amongst inhibitory neurons in a E-I network plays a pivotal role in controlling rhythmic PING-like dynamics. Changes to this connectivity can cause the inhibitory network to display dynamics beyond the single burst per oscillatory cycle typically seen in PING rhythms. By analyzing networks that do not satisfy this largely artificial constraint, we reveal that changing dynamics amongst the inhibitory cells are the impetus behind patterns formed in the excitatory cells that diverge from the classic PING predictions. Changes in the dynamics of the excitatory network are of paramount importance in biological networks where the excitatory pyramidal cells serve to output the signal generated by an E-I network to other brain regions.

Such networks with strong I-I connectivity display behavior largely explained by the conceptual PING model. For example, the inability for these networks to exhibit any excitatory synchrony for low E-I synaptic weights follows directly from the classical PING theory (Kopell et al., 2010). In this case, the strong intra-connectivity between inhibitory cells, combined with a weak excitatory drive to the inhibitory cells due to the weak E-I synaptic weight, prevents enough net drive from accumulating in the inhibitory cells to elicit a synchronous burst. This parallels the behavior seen in strictly inhibitory networks with strong inhibitory connectivity, in which networks with a low average intrinsic cell firing frequency, analogous to the excitatory drive to the inhibitory cells seen here, exhibit complete asynchrony. This behavior is explained in greater detail in our previous work, as the synaptic weight values analyzed in those strictly inhibitory networks are in an analogous range to the strong inhibitory intra-connectivity in our Strong Network here (Rich et al., 2016).

Furthermore, in Strong Networks with increased E-I synaptic weight, which results in a stronger drive to the inhibitory cells, the strong inhibitory intraconnectivity ensures only a single inhibitory burst occurs in response to excitatory cell activity. This provides for nearly identical levels of inhibition to each excitatory cell, which leads to classic PING activity (Kopell et al., 2010).

However, networks with weak I-I connectivity display divergent dynamics that have not been thoroughly analyzed by existing PING literature. These networks exhibit synchrony amongst the excitatory cells for very weak values of the E-I connectivity, contrary to the conceptual PING model (Traub et al., 1997; Ermentrout and Kopell, 1998; Whittington et al., 2000; Kopell et al., 2010). When the I-I synaptic weight is weak, less inhibition accumulates as a result of inhibitory intra-connectivity; this means that less excitatory drive is required to elicit a synchronous burst of inhibitory cell activity, which in turn leads to an excitatory burst. Here again the parallels to strictly inhibitory networks with weak inhibitory connectivity are apparent, as such networks were able to synchronize for low average intrinsic cell firing frequencies for which strongly connected networks were asynchronous.

Additionally, as the E-I connectivity strength increases, Weak Networks exhibit inhibitory cell dynamics beyond the single synchronous burst typically seen in PING networks. These dynamics can include multiple inhibitory bursts, as well as asynchronous inhibitory firing. Furthermore, the inhibitory patterning may differ in response to each excitatory burst. While such networks can still exhibit synchronous activity amongst the excitatory cells, the inconsistency of the inhibitory cell activity combined with the heterogeneous connectivity between neuron populations will cause a loss of well-organized and consistent bursting in the excitatory cell population, a feature which is reflected in the Variability Measure but not the Synchrony Measure.

With high E-I connectivity strength, Weak Networks do exhibit excitatory bursts with low Variability Measure in some cases. In these instances multiple inhibitory bursts occur, but the profile of these bursts is consistent and well-organized in response to each excitatory burst. The behavior of E-I networks with multiple inhibitory bursts, in particular the differences in excitatory network dynamics seen in response to disorganized vs. well-organized patterns of multiple inhibitory bursts, is not investigated in detail by the conceptual PING model.

The dichotomy between networks we deem “strongly” intra-connected and “weakly” intra-connected is in fact a robust feature when the I-I connectivity is varied. Indeed, by varying the strength of inhibitory intra-connectivity two distinct regimes of activity are revealed: networks exhibiting “strong behavior” show a low Variability Measure for a vast majority of networks in which any form of excitatory synchrony is achieved, while networks exhibiting “weak behavior” show synchronous excitatory activity at significantly lower values of the E-I connectivity strength but also exhibit increased variability in the excitatory bursting patterns as the E-I connectivity strength increases. Our analysis of networks with various values of the I-I connectivity also reveals that these two types of dynamics correspond with features in other important network properties. While networks exhibiting strong behavior show a monotonic increase in their average excitatory burst frequency as the E-I connectivity strength increases, networks exhibiting weak behavior show an overall decrease in this frequency, albeit with some upticks in frequency corresponding to networks where well-organized bursting is recovered thanks to consistent patterning in the inhibitory population. Additionally, in the parameter regime studied here networks exhibiting strong behavior will only exhibit a 1:1 ratio between inhibitory and excitatory bursts, while networks exhibiting weak behavior can achieve 2:1 and 3:1 burst ratios.

Perhaps most interestingly, our results highlight that the patterning of inhibitory activity, as influenced heavily by the I-I connectivity strength, controls the consistency of excitatory burst rhythmicity. When the inhibitory bursting pattern is consistent following each excitatory burst, be that pattern a single burst of activity as is classic in PING activity or multiple inhibitory bursts as we saw occur in networks with weak I-I connectivity, well-organized and consistent excitatory cell bursting is common and rhythmicity is periodic. However, when the inhibitory bursting pattern varies in response to each excitatory burst, different magnitudes and profiles of inhibitory current are generated. When the inhibitory input to the excitatory neurons varies from burst to burst, this disrupts the ability for these cells to exhibit consistent organization, leading to increased rhythm variability. Indeed, the importance of I-I connectivity in controlling inhibitory dynamics plays a crucial role in preserving consistent excitatory bursting and rhythmicity, revealing both the role of the I-I connectivity and inhibitory patterning to be of more importance in E-I network dynamics than previous studies of PING-like dynamics indicate.

We note that some of the behaviors of E-I networks focused on in this work, including multiple bursts of the inhibitory network, have been identified in previous PING literature without a thorough analysis (Börgers and Kopell, 2005). This research shows that when E-I networks display patterns of inhibitory behavior slightly beyond the classic restrictions of PING, such as the requirement that the inhibitory network only be active once per oscillatory cycle, the effect on the dynamics of the excitatory network can be more salient than previously suggested.

Furthermore, the same work by Börgers and Kopell identifies a broad “suppression boundary” between a regime of strict PING rhythms and a regime of asynchrony of the inhibitory cells that is affected by the strength of the I-I connectivity (Börgers and Kopell, 2005). Thus, it stands to reason that networks that exhibit patterns of multiple inhibitory bursts that are messy or inconsistent, such as the examples shown in Figures 3C,G, may exist in the region of bistability between strict PING rhythms and complete asynchrony of the inhibitory cells identified by Börgers and Kopell; by this interpretation, one can contextualize our work as expounding upon the dynamics of E-I networks in this regime where E-I network behavior is neither strictly rhythmic nor strictly asynchronous.

Finally, we note that all E-I networks studied here tend to exhibit more asynchrony and higher variability for networks with a stronger external drive to the excitatory cells. A clear example of this at work is seen in the differences between the example raster plots in Figures 3E,G. This result fits the predictions of more analytical work done by Börgers et al. (2010).

We chose to focus our research on networks containing Type I interneurons given the evidence that fast-spiking, PV+ interneurons, which often display Type I properties (Ferguson et al., 2013), make up a majority of the interneuron population in various brain regions (Muller et al., 2006; Povysheva et al., 2008). Additionally, a majority of the computational studies analyzing PING rhythms utilize Type I interneurons. However, given the important role intrinsic cellular properties play in determining inhibitory dynamics (Rich et al., 2016), we simulated E-I networks with interneurons modeled as Type II neurons with and without an M-type adaptation current to see if cell type plays a similarly important role in E-I networks. Strictly inhibitory networks of such neurons did not exhibit significant changes in dynamics in response to changing the inhibitory intraconnectivity, unlike such networks containing Type I interneurons; as expected, neither did E-I networks with Type II interneurons. This result indicates that PING-style networks with Type II interneurons exhibit more consistent activity in response to changes in the I-I connectivity.

The dichotomy between the dynamics of E-I networks with weakly intra-connected Type I interneurons and strongly intra-connected Type I interneurons motivated the creation of a E-I network utilizing heterogeneity in the I-I connectivity. Various studies have shown that heterogeneities can be used in neural networks to improve the network's ability to exhibit features such as rate coding (Mejias and Longtin, 2012), gain control (Mejias and Longtin, 2014), synchrony (Kriener, 2012), and robust oscillations (Xie et al., 2011). While many of these studies look at E-I networks similar to the ones analyzed here, the heterogeneities studied are not in the I-I connectivity.

The Strong/Weak Network created in this study implements heterogeneity in the I-I coupling by creating two inhibitory subnetworks, one that is strongly intraconnected and one that is weakly intraconnected. Given the vast diversity in cellular properties amongst interneurons (Buhl et al., 1994; Gonchar and Burkhalter, 1997; Gibson et al., 1999; Beierlein et al., 2000, 2003; Klausberger et al., 2003; Barthó et al., 2004; Somogyi and Klausberger, 2005; Klausberger and Somogyi, 2008), heterogeneity in the strength of inhibitory intra-connectivity amongst a population of interneurons is likely. Furthermore, numerous studies have shown that interneurons tend to intra-connect preferentially to those exhibiting similar properties (Gibson et al., 1999; Beierlein et al., 2003; Klausberger et al., 2003; Somogyi and Klausberger, 2005; Wang et al., 2011), in a sense forming the “subnetworks” we model in the Strong/Weak Network. In addition, many of these same studies show evidence for these different types of interneurons connecting with the same excitatory pyramidal cells, forming a network similar to that modeled here. Thus, there is biological motivation for creating a network not only with heterogeneity amongst the interneuron intra-connectivity, but also with inhibitory subnetworks without interconnectivity that synapse onto the same excitatory cell population.

Indeed, the heterogenous network structure broadens the parameter regime in which well-organized and consistent excitatory bursting patterns are achieved. While Strong Networks did not achieve any sort of synchronous dynamics amongst excitatory cells for low values of the E-I connectivity, Strong/Weak Networks do. Additionally, while Weak Networks exhibited excitatory bursting without well-organized or consistent excitatory bursting for high values of the E-I synaptic weight, Strong/Weak Networks decrease the Variability Measure in this parameter regime significantly. Thus, our Strong/Weak Networks provide a potential mechanism by which PING rhythms might be generated more robustly for a variety of external drives to the excitatory cells and E-I synaptic weights.

## Author contributions

Simulations performed by SR. Analysis of results and articulations of underlying mechanisms performed by SR, VB, and MZ. Paper written and edited by SR, VB, and MZ.

### Conflict of interest statement

The authors declare that the research was conducted in the absence of any commercial or financial relationships that could be construed as a potential conflict of interest.
